# Differential transcriptomic responses to *Fusarium graminearum* infection in two barley quantitative trait loci associated with *Fusarium* head blight resistance

**DOI:** 10.1186/s12864-016-2716-0

**Published:** 2016-05-21

**Authors:** Yadong Huang, Lin Li, Kevin P. Smith, Gary J. Muehlbauer

**Affiliations:** Department of Agronomy and Plant Genetics, University of Minnesota, St. Paul, MN 55108 USA; Department of Plant Biology, University of Minnesota, St. Paul, MN 55108 USA

**Keywords:** *Hordeum vulgare*, Barley, *Fusarium* head blight, *Fusarium graminearum*, QTL, RNA-Seq

## Abstract

**Background:**

*Fusarium graminearum* causes *Fusarium* head blight (FHB), a major disease problem worldwide. Resistance to FHB is controlled by quantitative trait loci (QTL) of which two are located on barley chromosomes 2H bin8 and 6H bin7. The mechanisms of resistance mediated by FHB QTL are poorly defined.

**Results:**

Near-isogenic lines (NILs) carrying Chevron-derived resistant alleles for the two QTL were developed and exhibited FHB resistance in field trials. To understand the molecular responses associated with resistance, transcriptomes of the NILs and recurrent parents (M69 and Lacey) were investigated with RNA sequencing (RNA-Seq) after *F. graminearum* or mock inoculation. A total of 2083 FHB-responsive transcripts were detected and provide a gene expression atlas for the barley-*F. graminearum* interaction. Comparative analysis of the 2Hb8 resistant (R) NIL and M69 revealed that the 2Hb8 R NIL exhibited an elevated defense response in the absence of fungal infection and responded quicker than M69 upon fungal infection. The 6Hb7 R NIL displayed a more rapid induction of a set of defense genes than Lacey during the early stage of fungal infection. Overlap of differentially accumulated genes were identified between the two R NILs, suggesting that certain responses may represent basal resistance to *F. graminearum* and/or general biotic stress response and were expressed by both resistant genotypes. Long noncoding RNAs (lncRNAs) have emerged as potential key regulators of transcription. A total of 12,366 lncRNAs were identified, of which 604 were FHB responsive.

**Conclusions:**

The current transcriptomic analysis revealed differential responses conferred by two QTL during *F. graminearum* infection and identified genes and lncRNAs that were associated with FHB resistance.

**Electronic supplementary material:**

The online version of this article (doi:10.1186/s12864-016-2716-0) contains supplementary material, which is available to authorized users.

## Background

*Fusarium* head blight (FHB) of small grains is caused by fungal pathogens of *Fusarium* species, primarily *Fusarium graminearum*. FHB is widespread in wheat (*Triticum aestivum* L.) and barley (*Hordeum vulgare* L.) production areas globally and has caused significant economic losses due to reduced yield and grain quality [1, 2]. *F. graminearum* infects spikes and the resulting infected grains are often contaminated with trichothecene mycotoxins (such as deoxynivalenol, DON) and create adverse health issues for animal and human consumption [3]. Practices to manage FHB include deploying resistant varieties, crop rotation and fungicide application. Developing a thorough understanding of the defense response and genetic mechanisms conferring resistance to *F. graminearum* infection may result in more precise genetic manipulation and control of the disease.

*Fusarium* species infect barley after anthesis [4] and colonize the brush hairs (ovary epithelial hairs) at the extruded seed tip, followed by invasion into the developing caryopsis [5]. In contrast, *Fusarium* species infect wheat spikes during anthesis with colonization of floret surfaces first and followed by penetration of floral tissues and spread within the spike, ultimately resulting in bleaching of the whole spike [6–8]. Wheat exhibits two primary forms of partial resistance to FHB which are termed type I (resistance to initial infection) and type II (resistance to spread of infection) resistance [9]. In barley, disease symptoms do not spread in the spike, even in susceptible cultivars, indicating that barley exhibits a natural level type II resistance [4]. During the infection process, *F. graminearum* grows intercellularly and asymptomatically at the advancing hyphal front and later grows intracellularly and induces host cell death [10–12]. Virulence of *F. graminearum* on host cells is associated with the expression of genes encoding plant cell wall degradation enzymes (CWDEs), proteases, lipases and enzymes for trichothecene biosynthesis [13–15].

A number of studies have examined the host response in wheat and barley to *F. graminearum* infection or DON application via profiling the host transcriptome and identified host genes providing basal plant-pathogen resistance and genes responding specifically to trichothecene accumulation [16–22]. In general, host plants respond to pathogen attack with two forms of defense mechanisms. Conserved pathogen-associated molecular patterns (PAMPs, such as flagellin or chitin) or damage-associated molecular patterns (DAMPs, such as oligogalacturonides or peptides) are perceived by plant pattern recognition receptors (PRRs) and result in PAMP-triggered immunity (PTI). To counteract PTI, adapted pathogens deliver effector proteins to enhance virulence which can be recognized by intracellular resistance proteins (R proteins) and initiate effector-triggered immunity (ETI) [23, 24]. Downstream events of PTI and ETI include calcium ion influx, generation of reactive oxygen species (ROS), signaling through kinase cascades, hormonal changes, transcriptional reprogramming, cell wall appositions and hypersensitive response. In addition to general defense strategies, barley genes that specifically respond to trichothecenes have been identified. It has been shown that a DON-inducible uridine diphosphate glucosyltransferase gene (*HvUGT13248*, MLOC_65675) can convert DON to less toxic DON-3-O-glucoside (D3G) in yeast [25]. Transgenic Arabidopsis and wheat expressing *HvUGT13248* exhibited increased resistance to DON [26, 27]. In addition, ABC transporters and glutathione-S-transferases (GSTs) have been implicated in DON tolerance [16, 28]. Recently, long noncoding RNAs (lncRNAs) have been shown to act as potential regulators of transcriptional response to pathogen infection. A study identified *F. oxysporum* responsive lncRNAs in *Arabidopsis thaliana* and suggested that lncRNAs were important components of the antifungal network [29].

In barley, resistance to FHB is partial and conditioned by quantitative trait loci (QTL). Two major QTL were identified on chromosome 2H bin8 (2Hb8) and 6H bin7 (6Hb7) in the six-rowed cultivar Chevron [30–32]. The resistance allele at the 2Hb8 QTL was associated with late heading date and a fine mapping study showed that heading date and FHB resistance were controlled by tightly linked loci [33]. FHB resistance at the 6Hb7 QTL was associated with high grain protein content and it is not known if this is due to linkage or pleiotropy [34]. Although numerous QTL have been identified, the understanding of the genetic mechanisms that these QTL contribute to resistance is limited. To determine the responses associated with FHB resistance conferred by the 2Hb8 and 6Hb7 QTL, we performed transcriptomic profiling using RNA-Seq on two R NILs and their susceptible recurrent parents M69 and Lacey, respectively. The objectives of this study were to (1) develop an atlas of gene expression in barley during *F. graminearum* infection; (2) identify transcriptional differences between the resistant and susceptible alleles at both QTL and discover potential defense mechanisms to FHB; and (3) identify and characterize lncRNAs during barley-*Fusarium* interaction. Our results provide the foundation for examining the mechanisms of FHB resistance conferred by two resistant QTL.

## Results

### Genetic and phenotypic characterization of the chromosome 2H bin8 and 6H bin7 FHB QTL NILs

Two FHB resistant alleles derived from Chevron, 2Hb8 and 6Hb7, were introgressed via backcrossing into the susceptible genotypes M69 (a breeding line) and Lacey (a cultivar), respectively. To map the introgressed regions, the plants carrying the resistant allele at 2Hb8 and 6Hb7 and the susceptible recurrent parents were genotyped with the barley 9 K iSelect SNP array [35] and the results revealed that 94.2 and 93.9 % of the recurrent parent genome was recovered in the 2Hb8 resistant (R) NIL and 6Hb7 R NIL, respectively. Based on the iSelect consensus genetic map [36], the Chevron alleles were introgressed in the expected QTL regions (Additional file 1: Figure S1). The 2Hb8 QTL ranges from 43.27 cM (marker SCRI_RS_140819) to 69.55 cM (marker SCRI_RS_237688) on chromosome 2H, while the 6Hb7 QTL ranges from 38.12 cM (marker BOPA1_885-104) to 60.71 cM (marker SCRI_RS_206797) on chromosome 6H.

**Fig. 1:**
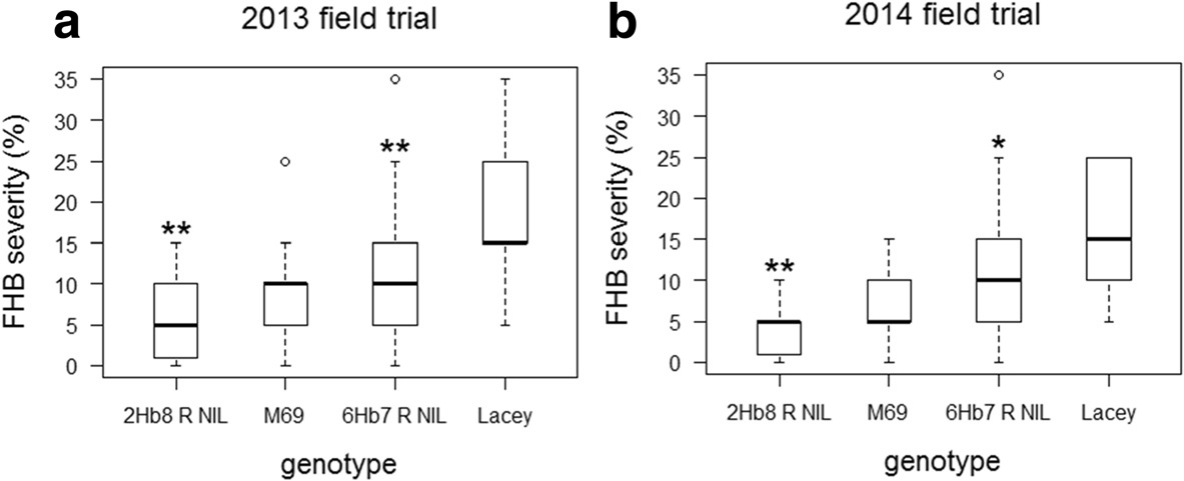
Disease resistance phenotype of the two FHB R NILs. The R NILs exhibited reduced FHB disease severity when compared to their respective recurrent parents in 2013 (**a**) and 2014 (**b**) field trials. Disease scores of each genotype are shown in box plots. *, *p* < 0.05, **, *p* <0.01

To evaluate the disease phenotypes (FHB severity) of the R NILs, replicated field trials were conducted in St. Paul, MN in 2013 and 2014. In both trials, the NILs exhibited significantly lower disease severity when compared to M69 and Lacey, respectively (Fig. 1). DON content in harvested grains was previously shown to be positively correlated with FHB severity [37, 38]. However, DON and ergosterol (an indication of fungal biomass) concentration during disease progression has not been investigated. To determine the pattern of DON and ergosterol accumulation in the NILs, we sampled the spikes from 8, 12, 16, 20 and 24 days post inoculation (dpi). As the disease progressed, both R NILs accumulated less DON and ergosterol when compared with their respective recurrent parents (Additional file 1: Figure S2). The difference in DON accumulation was significant at 8 dpi (2014), 12 dpi (2013) and 24 dpi (2014) for the 2Hb8 NIL and at 20 dpi (2014) for the 6Hb7 NIL. The difference in ergosterol accumulation was significant at 12 dpi (2013) for the 2Hb8 NIL and M69 comparison but there was no difference in the 6Hb7 NIL and Lacey comparison (Additional file 1: Figure S2). In addition, the contents of DON and ergosterol exhibited significant correlation within each genotype and year (Additional file 2: Table S1). Taken together, when compared to the susceptible parents these results indicated that the two R NILs exhibited reduced severity likely due to restricting fungal growth and DON accumulation.

### Transcriptome analysis

To examine the defense response and to gain an increased understanding of the genetic basis of FHB resistance in barley, RNA-Seq was performed to measure the transcriptome changes on three biological replications of the two NILs and their respective recurrent parents after *F. graminearum* or mock inoculation at 48 and 96 h after inoculation (hai). The experimental procedure and analysis pipeline are shown in Additional file 1: Figure S3. The accumulation of DON and ergosterol were analyzed for all samples used for sequencing (Additional file 3: Table S2). No DON or ergosterol was detected in any of the genotypes at 48 hai after mock or fungal inoculation. At 96 hai, the 2Hb8 R NIL accumulated less DON and ergosterol than M69 although the difference was not significant. Jia et al. [39] analyzed the 2Hb8 R NIL and M69 and observed significant difference in DON content at 96 hai. The 6Hb7 R NIL and Lacey samples had similar amounts of DON and ergosterol.

A total of 1.4 billion quality reads were generated from the 48 samples with an average of 29 million filtered reads per sample (Additional file 1: Figure S4, Additional file 4: Table S3). The RNA-Seq reads were mapped to the barley genome assembly [40] and between 15.6 million and 27.5 million uniquely-mapped reads were identified in the samples. Pearson correlation coefficients ranged from 0.72 to 0.99 between biological replicates (Additional file 1: Figure S5). Transcripts with differential expression levels (represented by FPKM values) were identified and referred to as differentially expressed genes (DEGs) (see methods and materials for definition). Expression levels of transcripts in *Fusarium*-inoculated samples were compared with those in mock-inoculated samples of the same genotype at each time point to identify *Fusarium* responsive DEGs. To identify DEGs associated with FHB resistance, expression levels of transcripts in the two R NILs were compared with the corresponding susceptible parents. Table 1 lists the number of DEGs identified in all pairwise comparisons (see Additional file 5: Table S4 for lists of transcripts and associated annotations). The RNA-Seq results were validated on 13 genes and one lncRNA for the two NILs and recurrent parents with qRT-PCR assays (*R*^2^ = 0.84, Additional file 6: Table S5 and Additional file 1: Figure S6).

**Table 1:** Number of differentially expressed genes (DEGs) identified by all pairwise comparisons

	RNA-Seq datasets comparison	number of DEGs			
		48 hai		96 hai	
		up	down	up	down
2Hb8	R NIL *Fusarium* inoculated – R NIL mock inoculated (R_F/M)	11	21	415	192
	M69 *Fusarium* inoculated – M69 mock inoculated (M69_F/M)	45	167	1283	354
	R NIL *Fusarium* inoculated – M69 *Fusarium* inoculated (R/M69_F)	350	80	321	426
	R NIL mock inoculated – M69 mock inoculated (R/M69_M)	355	70	401	282
6Hb7	R NIL *Fusarium* inoculated – R NIL mock inoculated (R_F/M)	100	14	304	15
	Lacey *Fusarium* inoculated – Lacey mock inoculated (Lacey_F/M)	12	53	693	227
	R NIL *Fusarium* inoculated – Lacey *Fusarium* inoculated (R/Lacey_F)	66	36	5	10
	R NIL mock inoculated – Lacey mock inoculated (R/Lacey_M)	12	89	4	19

### Barley host response to *F. graminearum* infection

**Fig. 2:**
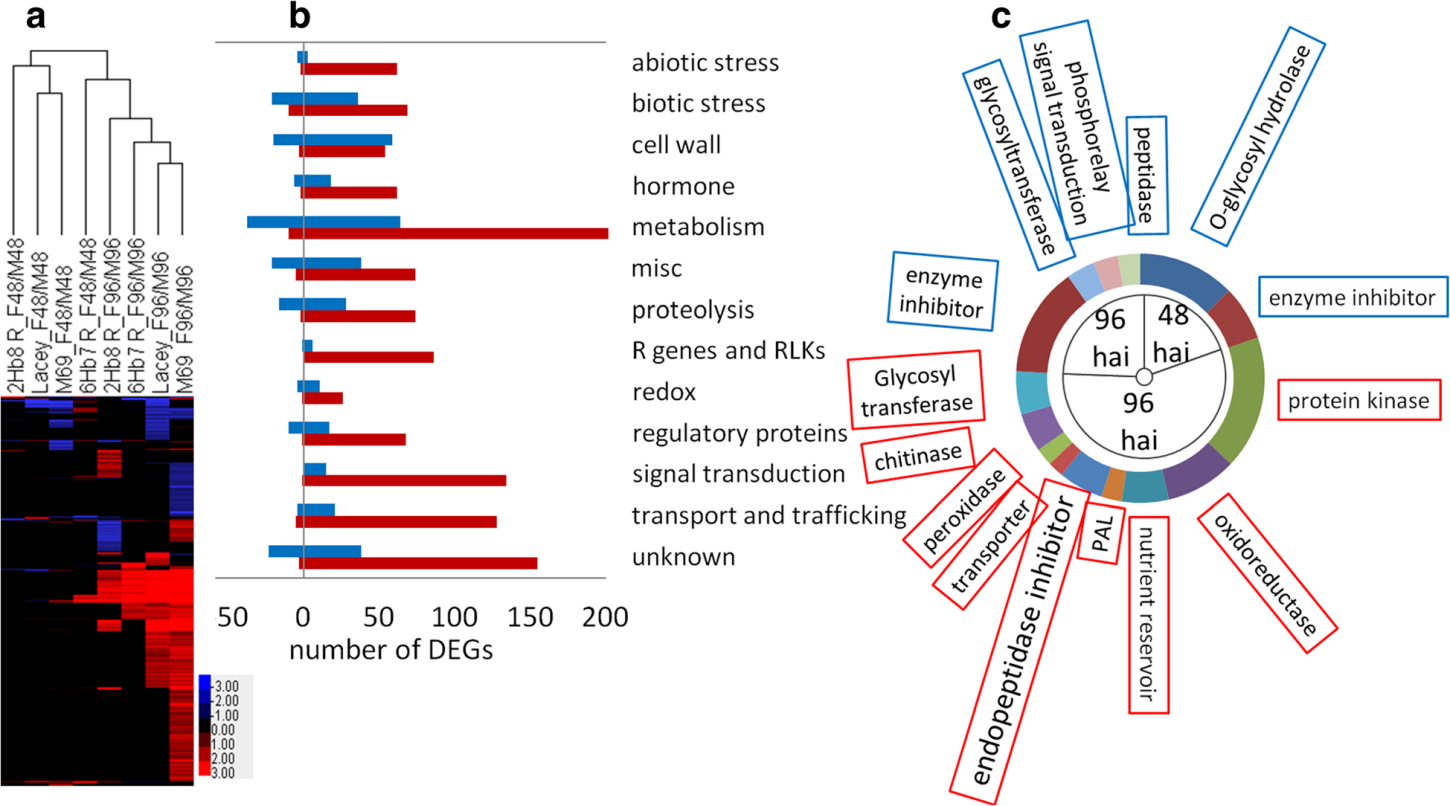
Barley host response to *F. graminearum* infection. **a** Hierarchical clustering of FHB-responsive differentially expressed genes (DEGs) in 2Hb8 R NIL, 6Hb7 R NIL, M69 and Lacey at 48 and 96 hai. F48/M48 indicates comparison of *F. graminearum*- versus mock-inoculated samples. Red, induced; blue, repressed. **b** Categorization of FHB induced (*red*) or repressed (*blue*) genes in M69 at 48 and 96 hai. **c** A sunburst plot of enriched GO terms identified in DEGs in M69 at 48 and 96 hai. Size of individual slices represent statistical significance (−log_10_FDR, larger size indicates higher significance). GO terms in blue boxes were enriched in repressed genes and GO terms in red boxed were enriched in induced genes. PAL, phenylalanine ammonia-lyase

The number of *Fusarium* responsive DEGs identified in the 2Hb8 R NIL – M69 and 6Hb7 R NIL – Lacey comparisons at 48 and/or 96 hai were 1935 and 1006, respectively. When combined, a unique set of 2083 FHB responsive DEGs were identified in the four genotypes after *F. graminearum* and mock treatments at 48 or 96 hai (Table 1 and Additional file 5: Table S4), representing 8.6 % of the high confidence genes (24,243) positioned on the Morex genome assembly [40]. Gene expression clustering based on *Fusarium* responsive DEGs revealed distinct groups of host genes responding to pathogen attack at 48 and 96 hai (Fig. 2a). For each genotype, the number of FHB responsive DEGs increased dramatically from 48 to 96 hai, indicating an increased defense response to fungal infection from early to late stage.

To gain a further understanding of the barley host response to *Fusarium* infection, the transcriptomic changes of the susceptible parent M69 after *F. graminearum* and mock inoculation were analyzed. Compared to mock inoculation, there were 46 and 1283 genes up-regulated and 167 and 354 genes down-regulated at 48 and 96 h after *F. graminearum* inoculation, respectively. These results demonstrate a dramatic increase in DEGs between 48 and 96 hai. For ease of interpretation, all the DEGs were categorized into broad functional classes based on their annotations which included abiotic stress (such as heat shock proteins), biotic stress (such as pathogenesis-related proteins), cell wall (such as cellulose synthases, chitinases and pectinesterase inhibitors), hormone, metabolism (primary and secondary), miscellaneous (proteins related to cell cycle, chromatin, retrotransposon, nodulin, scaffolding and protein-protein interaction), proteolysis (proteases, protease inhibitors and ubiquitination), disease resistance (R) genes and receptor-like kinases (RLKs), redox proteins (such as peroxidases and glutaredoxins), regulatory proteins (transcription factors), signal transduction (kinases and phosphatases), transport and trafficking, and unknown. (Fig. 2b, Additional file 5: Table S4).

An overrepresentation analysis using agriGO [41] was performed to identify enriched Gene Ontology (GO) terms associated with the barley-*F. graminearum* interaction in M69 (Fig. 2c and Additional file 7: Table S6). At 48 hai no enriched GO terms were identified for the up-regulated DEGs whereas two terms, O-glycosyl hydrolase activity (GO:0004553) and enzyme inhibitor activity (GO:0004857), were enriched in the down-regulated DEGs. The O-glycosyl hydrolase term includes gene products involved in carbohydrate metabolism, such as endoglucanases, glucosidases, invertases and galactosidases. The term enzyme inhibitor activity comprises endopeptidase inhibitors and pectin methylesterase inhibitors (PMEIs). At 96 hai nine functional groups were enriched in up-regulated DEGs, including kinases (GO:0004672), oxidoreductases (GO:0016491, such as laccases, 12-oxo-phytodienoic acid reductase (OPR) and aminocyclopropanecarboxylate (ACC) oxidase), storage proteins (GO:0045735, such as germin-like proteins), phenylalanine ammonia-lyases (GO:0016841, the first enzyme in phenylpropanoid pathway), serine-type endopeptidase inhibitors (GO:0004867, such as Bowman-Birk type trypsin inhibitors), transporters (GO:0005215), peroxidases (GO:0004601), chitinases (GO:0004568) and glycosyltransferases (GO:0016757). In contrast, four types of genes were enriched in down-regulated DEGs, including enzyme inhibitors (GO:0004857), glycosyltransferases (GO:0016757), peptidases (GO:0008233) and phosphorelay signal transduction system (GO:0000160, cytokinin signaling). MapMan analysis was performed to visualize DEGs involved in diverse metabolic pathways [42] (Additional file 1: Figure S7). These results suggested that at 96 hai there were up-regulation of pathways involved in amino acid and lipid metabolism, glutathione homeostasis, secondary metabolism and cell wall reinforcement. Pectin enzymes and lipid transfer proteins (LTPs) exhibited continuous down-regulation at both time points. Functional classification and GO enrichment analysis of *Fusarium* responsive DEGs in Lacey revealed similar results as those in M69 (Additional file 1: Figure S8 and Additional file 8: Table S7).

### Constitutive and induced defense response in the 2Hb8 R NIL to *F. graminearum* infection

**Fig. 3:**
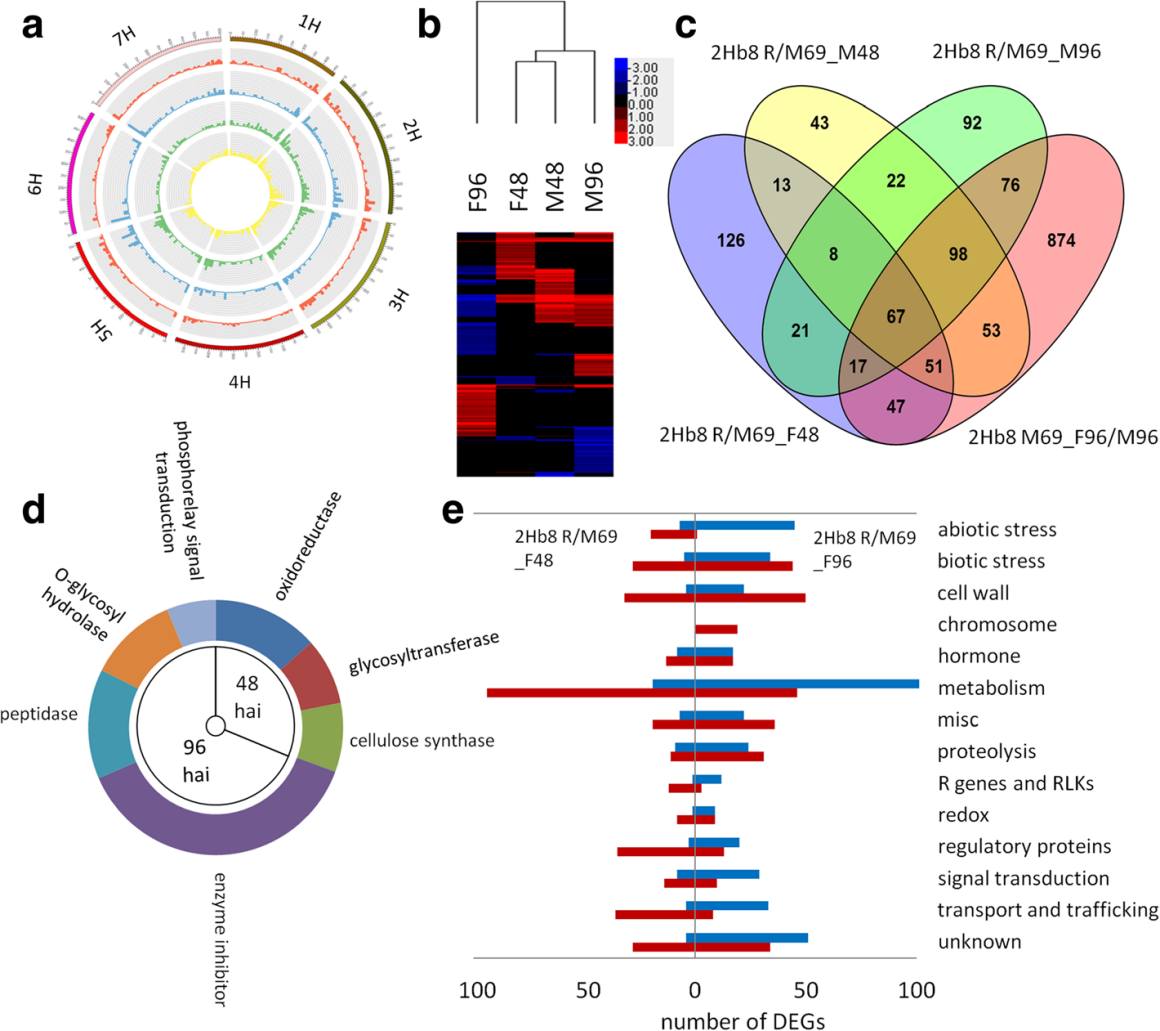
Constitutive and induced defense response conferred by the 2Hb8 QTL to *F. graminearum* infection. **a** Genome-wide distribution of DEGs between the 2Hb8 R NIL and M69. The outmost circle represents barley chromosomes 1H-7H. The height of histogram bins represents the number of DEGs in a defined genomic region. Tracks from inside going outside represents DEGs identified in comparisons 2Hb8 R/M69_M96 (*yellow*), R/M69_M48 (*green*), R/M69_F96 (*blue*) and R/M69_F48 (*red*). **b** Clustering of DEGs revealed constitutive elevation of defense response in the 2Hb8 R NIL when compared to M69. The 2Hb8 R NIL exhibited increased accumulation of transcripts after mock inoculation at 48 h (M48) and 96 h (M96) or fungal inoculation at 48 h (F48), which accumulated higher in M69 at 96 h (F96). The red color indicates induction and blue color repression. **c** Venn diagram of up-regulated genes in comparisons of 2Hb8 R/M69_F48, 2Hb8 R/M69_M48, 2Hb8 R/M69_M96 and M69_F96/M96. **d** A sunburst plot of enriched GO terms identified in up-regulated DEGs in the 2Hb8 R NIL compared to M69 at 48 and 96 hai. Size of individual slices represent statistical significance (−log_10_FDR). **e** Categorization of induced (*red*) or repressed (*blue*) genes in the 2Hb8 R NIL compared to M69 at 48 and 96 hai

To understand the FHB resistance responses mediated by the 2Hb8 QTL, transcriptomic comparisons were conducted between the 2Hb8 R NIL and M69 inoculated with *F. graminearum* or water (Table 1). In these comparisons, up-regulation means that transcript accumulation was higher in the R NIL, and down-regulation means that transcript accumulation was lower in the R NIL. Genomic positions of the DEGs in the four comparisons (2Hb8 R/M69_F48, 2Hb8 R/M69_F96, 2Hb8 R/M69_M48 and 2Hb8 R/M69_M96) showed a similar pattern of distribution as that of all high-confidence genes across barley chromosomes (Pearson correlation, *p < 2.2 e-16)* (Fig. 3a). Hierarchical clustering of the DEGs revealed two patterns (Fig. 3b). Firstly, the up-regulated gene profile of 2Hb8 R/M69_F48 showed the highest correlation with that of 2Hb8 R/M69_M48. A total of 139 genes were expressed higher in the R NIL both in the absence (mock treatment) and presence of *F. graminearum* infection at 48 hai (Additional file 9: Table S8). Secondly, 201 of the up-regulated genes in the 2Hb8 R/M69_M48 comparison exhibited down-regulation in the 2Hb8 R/M69_F96 comparison (Additional file 10: Table S9). Venn diagram analysis of up-regulated genes among comparisons 2Hb8 R/M69_F48, 2Hb8 R/M69_M48, 2Hb8 R/M69_M96 and M69_F96/M96 revealed that 52 % (182) of up-regulated genes in comparison 2Hb8 R/M69_F48 were induced later in M69 at 96 hai (Fig. 3c and Additional file 11: Table S10). Taken together, these results suggested that the 2Hb8 R NIL exhibited elevated defense responses in the absence of pathogen challenge (constitutive defense) and mounted a quicker defense response when challenged with *F. graminearum*.

**Fig. 4:**
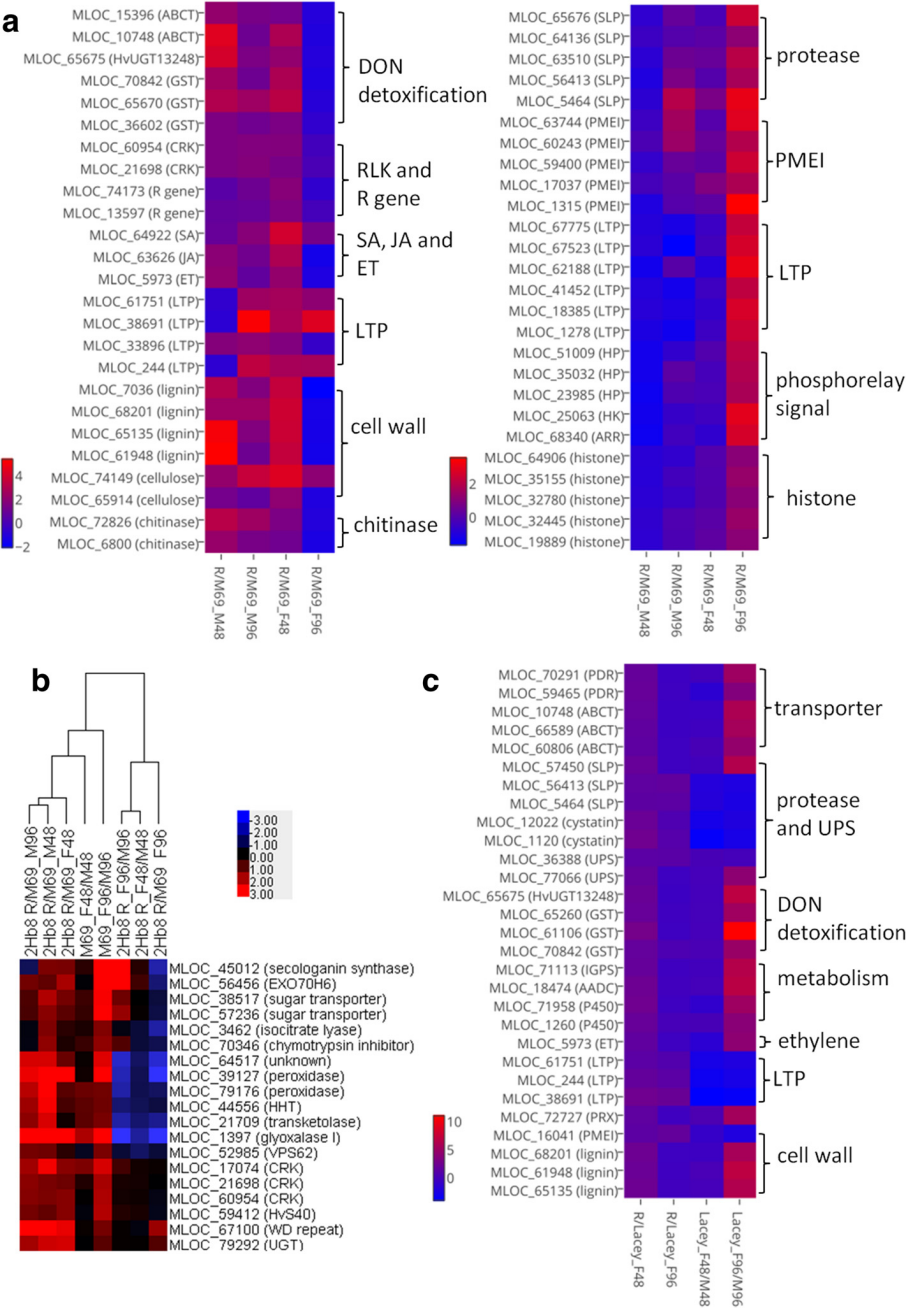
Representative genes associated with FHB resistance identified in the 2Hb8 R NIL and 6Hb7 R NIL after *F. graminearum* infection. **a** Left panel, representative genes that had increased accumulation in the 2Hb8 R NIL compared to M69 at 48 hai. Right panel, representative genes that had increased accumulation in the 2Hb8 R NIL compared to M69 at 96 hai. ABCT, ABC transporter; GST, glutathione-S-transferase; CRK, cysteine-rich receptor-like kinase; LTP, lipid transfer protein; SLP, subtilisin-like protease; PMEI, pectin methylesterase inhibitor; HP, histidine-containing phosphotransfer protein; HK, histidine kinase; ARR, two-component response regulator. **b** Expression profiles of candidate genes in the 2Hb8 QTL region. EXO70H6, exocyst subunit exo70 family protein H6; HvS40, senescence regulator S40; VPS62, vacuolar protein sorting-associated protein 62; HHT, omega-hydroxypalmitate O-feruloyl transferase. **c** Representative genes that had increased accumulation in the 6Hb7 R NIL compared to Lacey at 48 h after *F. graminearum* inoculation. Note the later induction of genes in Lacey at 96 h after *F. graminearum* inoculation. PDR, pleiotropic drug resistance protein; UPS, ubiquitin proteasome system; IGPS, indole-3-glycerol phosphate synthase; AADC, aromatic amino acid decarboxylase; PRX, peroxidase

To identify gene functions associated with FHB resistance mediated by the 2Hb8 QTL, GO enrichment analysis of up-regulated genes in comparisons of 2Hb8 R/M69_F48 and 2Hb8 R/M69_F96 were performed (Fig. 3d). Three GO terms were overrepresented in the comparison 2Hb8 R/M69_F48, namely, oxidoreductase activity (GO:0016491), glycosyltransferase activity (GO:0016757, such as HvUGT13248) and cellulose synthase activity (GO:0016759). The oxidoreductases included enzymes with multiple functions, such as lignification (laccases), cuticular wax production (WAX2-like), ethylene (ET) and jasmonic acid (JA) biosynthesis (ACC oxidase, OPR and lipoxygenase), secondary metabolism (alkaloids, phytoalexin and P450s) and peroxidases. Four terms were enriched in the comparison 2hb8 R/M69_F96, including enzyme inhibitor activity (GO:0004857, such as PMEIs), peptidase activity (GO:0008233), O-glycosyl hydrolase activity (GO:0004553) and phosphorelay signal transduction (GO:0000160). Functional categorization of DEGs (Fig. 3e) found that at 48 hai the 2Hb8 R NIL had more up-regulated genes involved in defense-related categories, such as R genes and RLKs, defense hormones (ET, JA and salicylic acid (SA)-related), peroxidases, cell wall fortification and stress responses (abiotic and biotic). At 96 hai, M69 exhibited an increased number of up-regulated genes in defense-related categories, suggesting that the timing of defense response in M69 was delayed when compared to the R NIL. The expression changes of representative defense transcripts at 48 and 96 hai are summarized in Fig. 4a.

Given the observed constitutive and induced elevation of defense responses in the R NIL and late, overlapping defense responses in M69, we hypothesized that the expression of candidate gene(s) for the 2Hb8 QTL might mirror the transcriptomic profiles of the R NIL and meet the following conditions: (1) they are located in the QTL region; and (2) they were expressed higher in the R NIL versus the susceptible parent after mock inoculation at 48 and/or 96 hai and/or after *F. graminearum* inoculation at 48 hai. We examined the expression profiles of nineteen genes that met these criteria (Fig. 4b and Additional file 12: Table S11). Three cysteine-rich receptor-like kinases (CRKs) appeared to be promising candidates (see discussion).

### Earlier induction of defense response mediated by 6Hb7 QTL

The expression profile of DEGs in the 6Hb7 R NIL at 48 hai clustered with those of all four genotypes at 96 hai, which indicated a quicker mobilization of defense response mediated by the Chevron allele at the 6Hb7 QTL (Fig. 2a). After *Fusarium* inoculation, a total of 100 genes were induced in the 6Hb7 R NIL at 48 h, of which 44 (44.0 %) were induced in Lacey at 96 hai (Additional file 13: Table S12). Comparisons of the resistant and susceptible genotypes at the 6Hb7 QTL after *F. graminearum* inoculation identified 102 and 15 transcripts with differential accumulation patterns at 48 hai and 96 hai, respectively. At 48 hai, transcripts with increased accumulation in the R NIL were involved in cell wall, biotic stress, proteolysis and transport (Additional file 1: Figure S9). Representative transcripts encoded defense-related proteins including HvUGT3248, PMEI, PR proteins, ACC synthase, GSTs, proteases, protease inhibitors and ABC transporters (Fig. 4c).

### Transcripts accumulated higher in both R NILs and expression of *HvUGT13248*

A group of 34 transcripts exhibited increased accumulation in the R NILs when compared to the respective susceptible parents at 48 hai (Additional file 14: Table S13). These transcripts encode proteins such as HvUGT13248, PR proteins, ACC synthase, cytochrome P450s, GSTs and transporters. This suggested that these transcripts might be involved in basal defense to *Fusarium* infection, and were co-regulated by two QTL. The *HvUGT13248* gene had increased accumulation in both 2Hb8 and 6Hb7 R NILs when compared to the respective susceptible parents at 48 hai. At 96 hai, expression of *HvUGT13248* in the R NILs and recurrent parents were induced to comparable levels. Barley genotypes, even ones susceptible to FHB, exhibit natural type II resistance to *F. graminearum* by delaying or restricting the spread of the disease in the spike and this resistance may be partially associated with the ability of HvUGT13248 to convert DON to D3G [25]. The higher accumulation of *HvUGT13248* during the early stage of fungal infection (within 48 hai) in both R NILs may further enhance resistance to *F. graminearum*.

### Identification of lncRNAs from barley spikes responsive to *F. graminearum* inoculation

Long noncoding RNAs (lncRNAs) have emerged as important regulators of transcription [43]. Genome-wide identification of lncRNAs from plants has been reported recently [44–46]. Applying the informatics pipeline developed by Li et al. (2014), we identified 12,366 lncRNAs from the four barley genotypes inoculated with *F. graminearum* or water at 48 and 96 hai (Additional file 1: Figure S10A and Additional file 15: Table S14). The class codes of lncRNAs describing their relationship with the barley reference transcripts were summarized in Additional file 16: Table S15. The majority (94.9 %) of lncRNAs were intergenic lncRNAs (lincRNAs). Thirty-four were long noncoding natural antisense transcripts (lncNATs). A comparison of lncRNAs to the barley high confidence protein–coding transcripts revealed that lncRNAs have fewer exons and shorter transcript length than protein-coding RNAs (Additional file 1: Figure S10B and C). Differentially expressed lincRNAs (DELs) were identified applying the same criteria as in DEGs analysis. We identified 449 and 284 FHB responsive DELs in the 2Hb8 R NIL-M69 comparison and the 6Hb7 R NIL-Lacey comparison, respectively. After removing duplicates, a total of 604 lincRNAs were FHB responsive in all genotypes at 48 and/or 96 hai. The number of differentially accumulated lincRNAs was 611 in the 2Hb8 R NIL compared to M69 and was 254 in the 6Hb7 R NIL compared to Lacey. The number of up- and down-regulated lincRNAs in respective comparisons is listed in Additional file 17: Table S16 and the associated lncRNAs are listed in Additional file 18: Table S17.

**Fig. 5:**
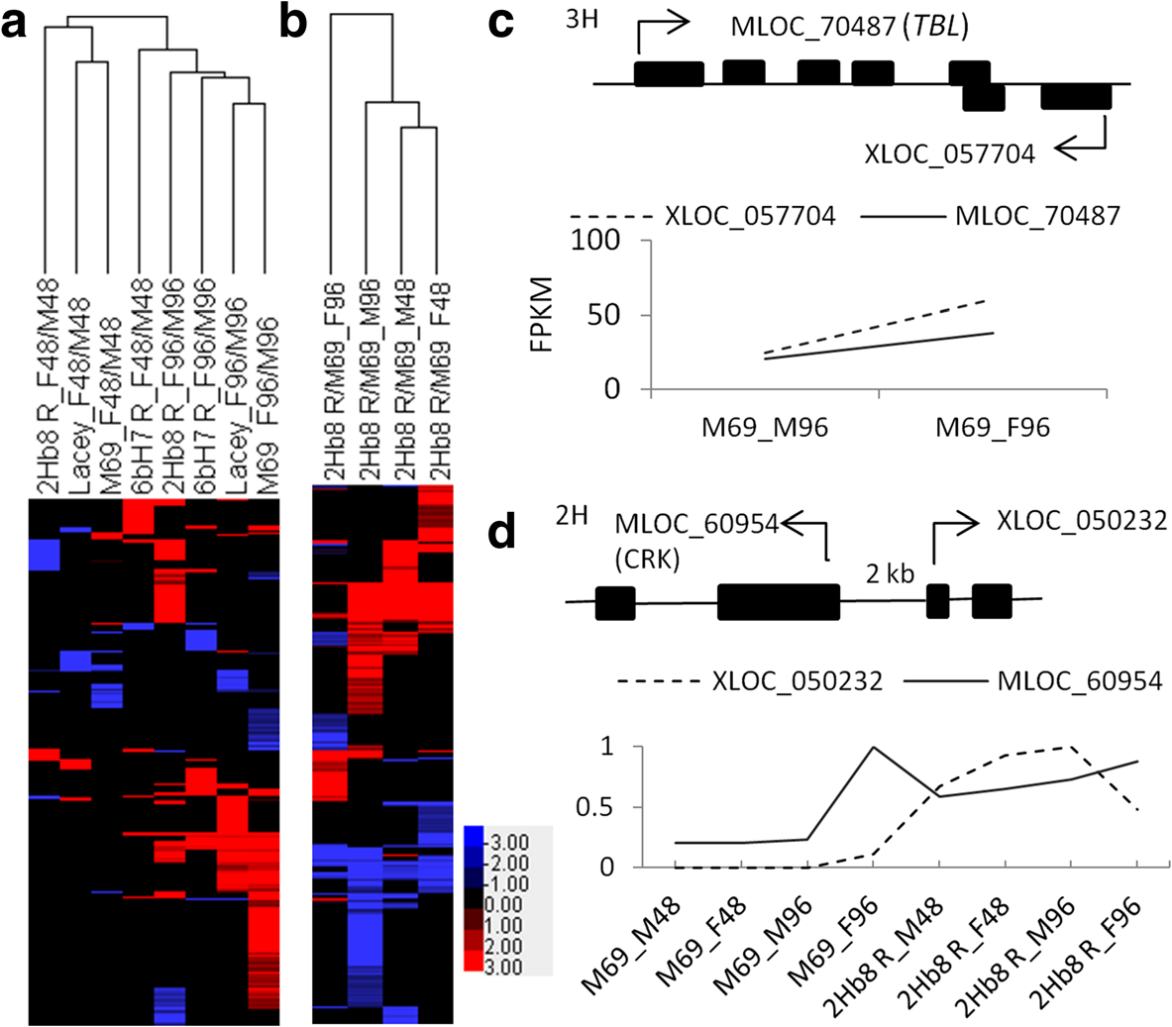
Clustering of differentially expressed lncRNAs (DELs) in the NILs and recurrent parents. **a** FHB responsive DELs in the NILs at 48 and 96 hai. **b** Constitutive expression of DELs in the 2Hb8 R NIL compared to M69. The 2Hb8 R NIL exhibited increased accumulation of a subset of lncRNAs after mock inoculation at 48 and 96 h or fungal inoculation at 48 h. **c** Co-regulation of an lncNAT/mRNA pair. XLOC_057704 and its sense transcript (*TBL)* showed co-induction in M69 at 96 h after *F. graminearum* inoculation. **d** Positive correlation of the induction of a CRK and an upstream lncRNA. Expression value (FPKM) of MLOC_60954 in individual treatment was normalized to the maximum expression value of MLOC_60954 among all treatments which was set to 1 (y-axis). The same was applied to normalize the expression value of XLOC_050232

Hierarchical clustering of the FHB responsive lincRNAs identified that the expression profile of DELs in 6Hb7 R_F48/M48 clustered with those of the four genotypes at 96 hai (Fig. 5a). Clustering of DELs between 2Hb8 R NIL and M69 revealed that the profiles of DELs in the datasets 2Hb8 R/M69_F48 and 2Hb8 R/M69_M48 were most similar, indicating higher accumulation of FHB responsive DELs in the 2Hb8 R NIL in the absence of *Fusarium* attack (Fig. 5b).

### Co-induction of lncRNAs and neighboring transcripts

Transcription of lncRNAs can regulate the expression of neighboring genes [47]. By comparing the expression levels of lncNATs and their sense transcripts, we identified one lncNAT (XLOC_057704), which exhibited induction in the M69_F96/M96 comparison. Its sense transcript (MLOC_70487) which encoded a trichome birefringence-like (TBL) protein involved in cellulose biosynthesis [48] showed co-induction (1.8 fold, *q* = 7.4 × 10^−4^, verified by qPCR) (Fig. 5c and Additional file 6: Table S5). The lncNAT XLOC_057704 was transcribed from the 3’ end and overlapped with the last exon of its sense transcript. In addition, five lincRNAs exhibited co-induction with their nearby transcripts after fungal inoculation. These transcripts encoded two receptor-like kinases, an alpha/beta fold hydrolase, a late embryogenesis abundant protein and an unknown protein (DUF538) (Additional file 19: Table S18). One of the RLKs was a cysteine-rich receptor-like kinase (MLOC_60954), which was identified as a candidate gene for the 2Hb8 QTL. The lincRNA XLOC_050232 is located approximately 2 kb upstream of MLOC_60954 (Fig. 5d). Its expression was not detected in M69 after mock inoculation (48 and 96 hai) or *F. graminearum* inoculation at 48 hai but was induced after *F. graminearum* inoculation at 96 hai, which coincided with the induction of MLOC_60954 in M69. In the 2Hb8 R NIL, XLOC_050232 was constitutively expressed in mock or *F. graminearum* inoculated samples at both 48 and 96 hai which coincided with the constitutively higher accumulation of MLOC_60954 in the 2Hb8 R NIL compared with M69 (Fig. 5d).

## Discussion

### Gene expression atlas of barley host responses to *F. graminearum* infection

In the present study we investigated the defense response of two barley NILs and their recurrent parents to *F. graminearum* infection. Previous transcriptomic analyses of barley-*F. graminearum* were conducted using the barley1 GeneChip technology and provided a framework for understanding the complex interaction of the pathosystem [16, 20, 21, 39]. Next-generation sequencing technology provides more sensitivity and versatility in querying the transcriptome changes than microarray technologies [49]. In addition, the assembly of the barley genome allowed genome-wide transcriptional profiling using RNA-Seq technology. We identified 2083 FHB responsive genes in four barley genotypes of which 248 (53 %) and 225 (47 %) genes were also identified by Boddu et al. [20] and Boddu et al. [21], respectively, validating the reliability and reproducibility of our results. The high sensitivity of RNA-Seq permitted detection of more DEGs in certain categories. For example, 74 and 9 receptor-like kinase transcripts were identified by this study and Boddu et al. [20], respectively. In addition, transcripts involved in new functional groups were discovered, such as pectinesterase inhibitors, cuticular wax biosynthesis, small auxin up RNAs (SAURs), gibberellin biosynthesis and phosphorelay signal transduction. The significantly increased volume of data provided by RNA-Seq enabled us to conduct hierarchical clustering and enrichment analysis to better understand the host response to *Fusarium* infection and potential molecular mechanisms conferred by resistant RILs. LncRNAs have been shown to play important roles in regulating transcription. However, the identification and characterization of lncRNAs in barley-*Fusarium* interaction have not been reported. We identified lncRNAs from the barley RNA-Seq dataset and analyzed their differential expression patterns in response to *Fusarium* infection. Co-regulation of lncRNAs and mRNAs were also uncovered. Thus, our RNA-Seq results of barley- *F. graminearum* interaction provide a comprehensive gene expression atlas for gene and lncRNA discovery and further comparative studies of wheat-*Fusarium* interaction or other cereal-pathogen pathosystems.

### Constitutive and induced defense response mediated by the 2Hb8 QTL

**Fig. 6:**
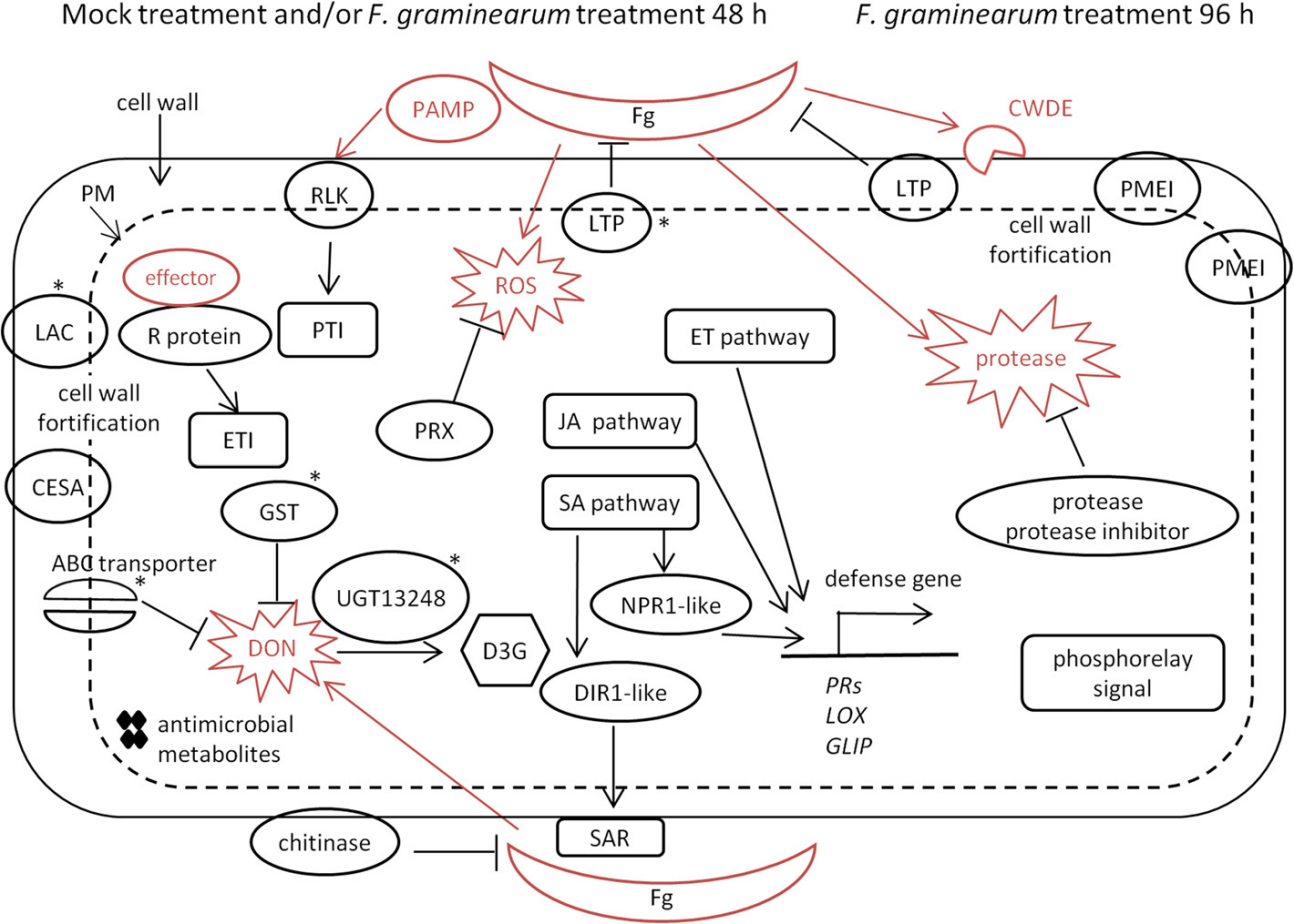
A proposed working model for FHB resistance conferred by the barley 2Hb8 QTL. Note the higher expression of defense-related genes in the 2Hb8 R NIL when compared to M69 during the mock treatment and within 48 h after *F. graminearum* infection. Asterisks indicate that the transcripts also accumulated higher in the 6Hb7 R NIL compared to Lacey after fungal infection at 48 h. Arrows indicate positive interaction. Perpendicular lines indicate negative interaction. Red color indicates virulence factor or signals derived from from *F. graminearum*. LAC, laccase; CESA, cellulose synthase; ROS, reactive oxygen species; NPR1, nonexpressor of PR genes 1; DIR1, defective in induced resistance 1; PR, pathogenesis-related; LOX, lipoxygenase; GLIP, GDSL lipase; PM, plasma membrane. See text for other abbreviations

The 2Hb8 QTL is considered a major locus for resistance to FHB and DON accumulation. A previous study examining the 2hb8 NIL pair identified 85 DEGs in the 2Hb8 R NIL compared to M69 after *F. graminearum* or water inoculation [39]. Due to the limited number of DEGs, it was not possible to perform clustering and enrichment analysis to identify patterns in differential transcription and develop an understanding of the gene expression dynamics of infection. In the present study, we identified 1481 transcripts that exhibited differential accumulation profiles in the 2hb8 R NIL compared with M69 after *F. graminearum* and/or mock inoculation at 48 and/or 96 hai. Our comparative transcriptomic analyses of the 2Hb8 R NIL and M69 suggested that the R NIL exhibited elevated expression of defense-related genes in the absence of *F. graminearum* challenge and mounted earlier and potentiated defense response when challenged with *F. graminearum*. A simplified model is proposed to elucidate the defense response provided by the 2Hb8 QTL (Fig. 6). At 48 h after fungal infection, nine RLKs and three NBS-LRR class R genes were expressed higher in the R NIL than in M69, indicating that the R NIL might recognize fungal elicitors and effectors and elicit PTI and ETI more efficiently. *F. graminearum* is classified as a hemibiotrophic pathogen, exhibiting a brief biotrophic phase during initial stage of infection before switching to necrotrophy and inducing cell death [13, 50]. Previous studies have suggested that the defense hormone SA contributed to wheat resistance to *F. graminearum* during early stage of infection and exhibited complex interaction with the JA pathway [18, 51]. The FHB resistance in wheat expressing Arabidopsis *NPR1* is associated with increased accumulation of SA and a systemic acquired resistance (SAR)-like response [51]. *NPR1* (MLOC_64922) and *DIR1* (MLOC_14218) are positive regulators of SA and SAR signaling, respectively [52] and we identified barley homologs of these genes that exhibited higher expression in the R NIL at 48 hai but not at 96 hai. We also identified elevated accumulation of *PR1* (an SA marker gene) transcripts in the R NIL compared to M69 after mock treatment. These results indicated that elevated SA/SAR signaling might be important for FHB resistance during the early stages of infection. It has been shown that JA and ET are involved in wheat and barley FHB resistance [53–55]. Genes in both biosynthesis and signaling pathways of JA and ET had increased accumulation in the R NIL compared to M69 at 48 h, such as *LIPOXYGENASE* (*LOX*) and *GDSL LIPASE* (*GLIP*). At 48 hai, the activities of oxidoreductases, cellulose synthases and glycosyltransferases were overrepresented in the R NIL, suggesting that the R NIL responded to the infection with cell wall reinforcement (such as lignification, cellulose synthesis, and cuticular wax production), expression of PR proteins (chitinases, lipid transfer proteins, defensins and thaumatin-like proteins) and antimicrobial metabolites (alkaloids and phytoalexin), and trichothecene detoxification (UGTs, GSTs and ABC transporters). The *HvUGT13248* (MLOC_65675) had higher expression in the R NIL at 48 h which could detoxify DON more rapidly and mitigate its toxic effects on barley cells.

As the disease progressed to 96 h, a shift in the gene categories was observed in the transcripts with higher accumulation in the R NIL. PMEIs, LTPs, proteases and phosphorelay signal transduction pathway were enriched in the R NIL (Fig. 6). In contrast, these activities were repressed in M69 after *F. graminearum* infection at both 48 and 96 h (Fig. 2c and Additional file 1: Figure S7). Overexpression of PMEI has been shown to limit fungal disease in Arabidopsis and wheat [56, 57]. Pathogen attack can induce PME activity and lead to decreased pectin methylesterification, which facilitates pathogen invasion of plant cells [58]. Our results suggested that *F. graminearum* might increase PME activity by repressing the expression of PMEI transcripts in M69, but in the R NIL the repression of PMEI transcripts may have been inhibited and cell wall pectin remained highly methylesterified, making the cell wall less susceptible to the effects of pectin degradation enzymes secreted by *F. graminearum*. Expression of genes encoding lipid transfer proteins can be induced by fungal pathogens. Biological roles of LTPs include antifungal activity, SAR signaling and cuticular lipid deposition [59]. Overexpression of a wheat LTP5 gene increased resistance to *Cochliobolus sativus* and *F. graminearum* [60]. An LTP gene is proposed as the candidate gene for the wheat FHB QTL Qfhs.ifa-5A [61]. And an Arabidopsis LTP enhances resistance to a trichothecene toxin by increasing glutathione content [62]. In the 2Hb8 R NIL compared to M69, after fungal infection the number of up-regulated LTP genes increased from 5 (48 hai) to 19 (96 hai), suggesting that they may contribute to the FHB resistance by inhibiting *F. graminearum* growth and/or reinforce the cuticle. During infection of host plants, *Fusarium* species secrete not only CWDEs, but also proteases to hydrolyze host proteins. Plants respond by producing their own proteases and protease inhibitors to inhibit fungal counterparts. Gottwald et al. [53] reported the enrichment of proteases and protease inhibitors in the transcriptome of the FHB resistant wheat cultivar after infection. We observed enrichment of proteases in the R NIL at 96 hai, including aspartyl proteases, subtilisin-like serine proteases and cysteine proteases. At 96 hai, the R NIL exhibited enrichment of genes involved in phosphorelay signal transduction pathway that was regulated by cytokinin. Eight genes functioning in cell cycle and division exhibited increased accumulation in the R NIL compared to M69 as well. One of the prominent roles of cytokinin is to control cell cycle and cell division. The up-regulation of the cytokinin pathway in the R NIL suggested that cell proliferation and kernel development were less disturbed in the R NIL than in M69, consistent with the reduced disease severity phenotype of the R NIL.

### Identification of candidate genes for the 2Hb8 QTL

Previous studies have suggested that higher accumulation of defense-related transcripts and activation of immunity in the absence of pathogen challenge contributed to disease resistance in soybean and Arabidopsis [63, 64]. Similarly, the 2Hb8 R NIL exhibited elevated expression of defense-related genes upon mock treatment. Based on physical location and expression profiles, three CRKs were identified as candidate genes for the QTL with two of them exhibiting high homology (MLOC_17074, MLOC_21698 and MLOC_60954, Additional file 1: Figure S11). A previous microarray analysis of the 2Hb8 NIL [39] identified a CRK gene (probe set name: rbaal11f18_at, which matches MLOC_21698) that exhibited similar expression profile as in this study. CRKs, also known as DUF26 (Domain of Unknown Function 26) RLKs, belong to a subfamily of RLKs and are characterized by two copies of the C-X8-C-*X*2-C motif in their extracellular domain [65]. Expression of CRKs can be induced by oxidative stress, SA and pathogen infection [66]. Overexpression of Arabidopsis CRKs conferred enhanced resistance to *Pseudomonas syringae* [67]. The CRK13 mediated resistance to *P. syringae* was dependent on SA accumulation, and transcriptomic analysis revealed significant overlap between CRK13 overexpression plants and wild-type plants treated with *P. syringae*. These results were concordant with our observations. The SA regulated barley homologs of *NPR1* and *DIR1* exhibited increased accumulation in the 2Hb8 R NIL compared to M69 at 48 h after fungal infection. Among the up-regulated genes in the R NIL versus M69 after mock treatment, 64.5 % (362 out of 561) were induced by *F. graminearum* infection in M69 at 96 hai. Given the function of Arabidopsis CRKs in disease resistance and our transcriptomic data, the three barley CRKs are promising candidates for the 2Hb8 QTL and merit further study.

### More rapidly induced early-stage defense responses conferred by the 6Hb7 QTL

The 6Hb7 QTL confers a minor effect on FHB resistance when compared to the 2Hb8 QTL and its transcriptomic response to *Fusarium* infection has not been studied. Our results indicated that the 6Hb7 R NIL exhibited an earlier induction of a set of genes involved in FHB response (Fig. 2a and Additional file 13: Table S12), suggesting a sensitized state to *F. graminearum* infection conferred by the QTL. Compared with the 2Hb8 R NIL and M69, the transcriptomic changes between the resistant and susceptible genotypes at the 6Hb7 QTL after *F. graminearum* inoculation were limited and occurred mainly during the early-stage of infection (48 hai). Among the 66 genes with increased accumulation in the 6Hb7 R NIL compared to Lacey after *F. graminearum* infection at 48 h, many encoded proteins which are associated with resistance to DON or *F. graminearum* (Fig. 6 and Additional file 5: Table S4). Walter et al. [28] reported that a wheat ABC transporter contributes to mycotoxin tolerance. We identified 3 ABC transporters with higher accumulation in the R NIL. The expression of *HvUGT13248* was higher in the 6Hb7 R NIL than in Lacey at 48 hai. However, we did not observe a reduced DON concentration in the 6Hb7 R NIL compared to Lacey at 96 hai (Additional file 3: Table S2). One possible explanation is that the inoculation method we used may overwhelm the resistance in the 6Hb7 R NIL. As a result, the two genotypes exhibited no difference in fungal biomass at 96 hai. We examined the 66 transcripts for candidate genes for the 6Hb7 QTL. Only one transcript was located within the QTL interval, which encoded a protein homologous to a maize F-box domain containing protein (MLOC_36388).

### Identification of lncRNAs and co-regulation of lncRNAs and mRNAs in response to *F. graminearum* infection

Recent years have witnessed discoveries of thousands of long noncoding RNAs (lncRNAs) in plant genomes and a few lncRNAs have been shown to regulate vernalization, male sterility and photomorphogenesis [68–70]. In addition, expression of lncRNAs can be altered by abiotic and biotic conditions, suggesting a role of lncRNAs in responding to stress [45, 71]. Zhu et al. [29] identified 20 *F. oxysporum* responsive intergenic lncRNAs (lincRNAs) of which five may play a role in resistance. We mined our RNA-Seq data to identify 12,366 lncRNAs from *F. graminearum* or water inoculated barley spike samples. Hierarchical clustering analyses revealed similar clustering patterns between differentially expressed lincRNAs (DELs) and DEGs (Fig. 2a and Fig. 5a), indicating that the transcriptional changes of lincRNAs and mRNAs are highly coordinated in barley-*F. graminearum* interactions.

Correlations between significantly regulated lncRNAs and mRNAs have been shown in Arabidopsis after *F. oxysporum* infection. In our study, one lncNAT (XLOC_057704) and its sense transcript (MLOC_70487) and five lincRNAs and their neighboring transcripts exhibited co-induction regulation after fungal infection. The lncNAT XLOC_057704 and MLOC_70487 were both induced in M69 after fungal infection at 96 hai. The MLOC_70847 encoded a trichome birefringence-like (TBR-like [TBL]) protein which was required for cellulose biosynthesis. The *tbr* and *tbl3* mutants had decreased cellulose and altered pectin composition [48]. Cellulose is important for cell wall apposition against pathogen attack and we observed enrichment of cellulose activity in the 2Hb8 R NIL compared to M69 at 48 hai. Thus, the induction of MLOC_70847 may have a role in disease resistance. It is unclear whether XLOC_057704 up-regulation has a causal relationship with the induction of MLOC_70847. Among the five lincRNAs and their proximal protein-coding transcripts that exhibited co-induction after fungal infection, one lincRNA (XLOC_050232) was located about 2 kb upstream of a CRK (MLOC_60954, a candidate gene for 2Hb8 QTL) gene on the same strand. The lincRNA/mRNA pair was transcribed in a divergent fashion. However, whether or how XLOC_050232 affected the transcription of the CRK is unknown and requires further research.

## Conclusions

The current RNA-Seq analyses of two barley NILs carrying resistant FHB QTL alleles revealed the molecular mechanisms of FHB resistance. The 2Hb8 QTL confers elevated defense response in the absence of *F. graminearum* infection, which enables the resistant NIL to mount an earlier and augmented defense response within 48 h of fungal infection. The transcript levels of the components involved in effector-triggered immunity and pattern-triggered immunity, as well as genes targeting *F. graminearum* virulence factors, such as DON, cell wall degrading enzymes and proteases, accumulated higher in the 2Hb8 resistant NIL when compared to M69. Based on expression profiles and function of homologous genes, three cysteine-rich RLK were identified as candidate genes for the 2Hb8 QTL. The 6Hb7 QTL mediates an earlier induction of defense genes within 48 h after fungal infection. These genes were involved in DON detoxification and cell wall fortification. In addition to mRNA profiling, lncRNAs were identified from barley spike samples and their differential expression in response to *F. graminearum* infection were characterized. Co-regulation of lncRNAs and neighboring mRNA suggests that their expression is highly coordinated in response to *F. graminearum* infection.

## Methods

### Plant materials, genotyping and FHB field trials

The 2Hb8 R NIL was developed by Nduulu et al. [33] through five backcrosses of a resistant F_4:7_ progeny from the Chevron/M69 mapping population carrying the Chevron haplotype for the 2Hb8 QTL region with the susceptible parent M69. The 6Hb7 R NIL was generated by backcrossing four times the Chevron allele at the 6Hb7 QTL region to a susceptible genotype Lacey [72]. M69 and Lacey are six-rowed spring barleys that were developed by the University of Minnesota barley breeding program. Leaf tissue from 2-week old seedlings was collected and genomic DNA of each genotype was extracted using the CTAB method. DNA samples were genotyped with the barley iSelect SNP array at the USDA-ARS in Fargo ND. Results of SNP analyses were visualized using the Spotfire software (TIBCO, Boston, MA).

Field trials of the genotypes were conducted in an FHB nursery at Saint Paul, MN in 2013 and 2014 following the methods of Nduulu et al. [33]. Seeds were planted in single rows 5–6’ long using a randomized complete block design with 5 replications. Plants were spray inoculated with a mixture of 39 local *F. graminearum* isolates twice (1 × 10^5^ spores/mL, 0.02 % Tween 20). The first inoculation was applied 2–3 days after heading and the second 3 days later. A mist irrigation system was applied to promote disease development. Plants were scored for FHB severity 2 weeks after the first inoculation by randomly selecting ten spikes per row and estimating the percentage of infected kernels within each spike. The difference in disease severity between the resistant and susceptible genotypes was tested by a Student’s *t* test. To determine the time course of DON accumulation in spikes, four or five spikes per row were randomly sampled, pooled and frozen to − 80 °C on the 8th, 12th, 16th, 20th and 24th day after the first inoculation. The pooled spikes per genotype/time point/replication were ground in liquid nitrogen to a fine powder and one gram of the powder was analyzed for DON and ergosterol content using gas chromatography–mass spectrometry [73]. The DON and ergosterol content were not analyzed for the 6Hb7 R NIL and Lacey (year 2013) due to insufficient number of samples.

### Growth chamber inoculation, sample collection and RNA sequencing

For the RNA-Seq experiments, a randomized complete block design with three biological replications was used. Four seeds of each genotype were planted in 5-inch pots filled with Sunshine MVP mix and plants were grown in a growth chamber (16 h light/8 h dark, 20 °C day/18 °C night). Inoculation was applied on barley spikes 2–3 days after their complete emergence from the boot. The spikes from the main culm of each plant were spray-inoculated with *F. graminearum* (strain PH-1) conidia suspension (1 × 10^5^ spores/mL, 0.02 % triton X-100) using an airbrush applicator (Paasche, Chicago, IL). For mock inoculation, conidia suspension was replaced with sterile water. Each side of the spike was sprayed twice from top to bottom and then covered in clear plastic bags which were removed at the time of sampling (48 and 96 hai). Four inoculated spikes per genotype/time point/treatment/replication were sampled, pooled and flash frozen in liquid nitrogen. A total of 48 samples were collected (four genotypes x two treatments x two time points x three biological replications). Total RNA was extracted from each sample using RNeasy Plant Mini Kit and on-column DNase digestion was performed to remove genomic DNA (QIAGEN, Valencia, CA). Sample QC, library creation and RNA sequencing (HiSeq 2000 platform, paired-end, 100 cycles) were performed at the University of Minnesota Genomics Center.

### RNA-Seq data analysis

The barley genome sequence data were retrieved from the barley FTP download page (ftp://ftpmips.helmholtz-muenchen.de/plants/barley/public_data/). All RNA-seq reads from each sample were trimmed by sequencing quality and mapped to barley reference genome using the spliced read aligner TopHat [74]. The parameters “-a 5 -m 1 -i 40 -g 1 --segment-mismatches 1 --microexon-search --coverage-search” as well as the high-quality gene annotation of barley reference genome as a guide were employed during the read mapping. Uniquely mapped reads were extracted to profile gene expression level (FPKM - normalized read number per gene) in each sample using cufflinks [75]. Differentially expressed genes (DEGs) or long noncoding RNAs were identified using Cuffdiff from the Cufflinks tool package with the reference genome high-confidence gene annotation. DEGs were represented by both quantitatively- and qualitatively-expressed genes. Quantitatively-expressed transcripts were defined as transcripts with a fold change in expression level greater than 2.0 and a false discovery rate (FDR) less than 0.05. Qualitatively expressed transcripts (presence or absence) were defined as those exhibiting an FPKM value greater than 10.0 versus a value of 0 in either *F. graminearum* or mock treatment. Correlation coefficients (*r*) of biological replicates were calculated and two replicates (6Hb7_R_M48_REP3 and 6Hb7_R_F96_REP2) were removed from further analysis due to low *r* values (Additional file 4: Table S3 and Additional file 1: Figure S5).

### Gene expression analysis

Barley high-confidence gene annotation files were downloaded from the barley FTP site. High-confidence genes without GO annotations were subjected to Blast2GO analysis [76]. GO terms were subject to singular enrichment analysis within agriGO [41]. Hierarchical Clustering of DEGs were performed using Cluster 3.0 [77] and visualized using Java Treeview [78]. Heat maps of DEGs were generated using Plotly (Montreal, Quebec). Visualization of metabolism-related DEGs were done using MapMan [42]. The experiment and mapping files were generated according to the manual. Venn diagrams were generated using Venny 2.0 [79].

### Quantitative RT-PCR validation

Total RNA were extracted from the same spike samples used for RNA-Seq using RNeasy Plant Mini Kit and DNase digestion (QIAGEN, Valencia, CA). RNA quality was assessed on a NanoDrop spectrophotometer (Thermo Scientific, Wilmington, DE) to ensure that all samples had A_260_/A_280_ and A_260_/A_230_ ratios greater than 2.0. First strand complementary DNA (cDNA) was synthesized from 0.5-1 μg total RNA using the ImProm-II reverse transcription system (Promega, Madison, WI). A set of transcripts with diverse roles in defense response were selected for qRT-PCR analysis. Gene-specific primers were designed to amplify 80–150 bp fragments that span exon-exon junctions or near the 3’ UTR region of the target gene. Q-PCR was performed using iTaq universal SYBR green supermix (Bio-Rad, Hercules, CA) on a StepOnePlus real time PCR system (Applied Biosystems, Carlsbad, CA) with the following protocol: 95 °C for 20 s, 40 cycles of 95 °C for 3 s and 60 °C for 30 s followed by a melt curve analysis. All reactions were run in triplicate and a barley tubulin gene (MLOC_60297) was used as an internal control. Results of qPCR reactions were analyzed using the comparative C_T_ method (ΔΔC_T_) [80] and the mock-treated recurrent parent was used as the calibrator. The genes selected for validation, time point, primer sequences and qPCR results were listed in Additional file 6: Table S5.

### Availability of supporting data

The RNA sequencing data were submitted to the NCBI database (BioProject ID PRJNA294716). The other supporting data were included as additional files.

## Additional files

Additional file 1: Figure S1. Graphical genotypes of the 2Hb8 R and 6Hb7 R NILs. **Figure S2**. The R NILs accumulated less DON and ergosterol than the respective susceptible genotypes M69 and Lacey. **Figure S3**. Flowchart of the experimental setup and RNA-Seq analysis. **Figure S4**. Number of RNA-Seq reads generated for each sample that were filtered and uniquely mapped. **Figure S5**. Correlation coefficients of biological replicates used in RNA-Seq experiments. **Figure S6**. Correlation of RNA-Seq and qRT-PCR results of 13 genes and one lncRNA. **Figure S7**. MapMan visualization of FHB-responsive DEGs involved in general metabolic pathways in M69 at 48 hai and 96 hai. **Figure S8**. Categorization of induced (red) or repressed (blue) genes in Lacey at 48 and 96 h after *F. graminearum* inoculation. **Figure S9**. Categorization of induced (red) or repressed (blue) genes in the 6Hb7 R NIL compared to Lacey at 48 and 96 h after *F. graminearum* inoculation. **Figure S10**. Identification and characterization of lncRNAs from barley spike samples. **Figure S11**. Amino acid sequence alignment of three cysteine-rich receptor-like kinases using Jalview. (DOCX 6130 kb) Additional file 2: Table S1. Pearson correlation coefficients between DON and ergosterol concentrations. (XLSX 11 kb) Additional file 3: Table S2. DON and ergosterol concentrations in the samples used for RNA-Seq. (DOCX 11 kb) Additional file 4: Table S3. Detailed information of RNA-Seq samples. (XLSX 11 kb) Additional file 5: Table S4. Lists of DEGs identified in pairwise comparisons in Table 1. (XLSX 734 kb) Additional file 6: Table S5. Primer sequences and results of qPCR validation of RNA-Seq experiments. (XLSX 12 kb) Additional file 7: Table S6. GO terms enriched in DEGs in susceptible parent M69 after *F. graminearum* inoculation. (DOCX 17 kb) Additional file 8: Table S7. GO terms enriched in DEGs in susceptible parent Lacey after *F. graminearum* inoculation. (DOCX 18 kb) Additional file 9: Table S8. List of 139 genes with higher expression levels in the 2Hb8 R NIL in both mock and fungal treatments compared to M69 at 48 hai. (XLSX 24 kb) Additional file 10: Table S9. List of 201 genes with higher expression levels in comparison 2Hb8 R/M69_M48 and lower expression in comparison 2Hb8 R/M69_F96. (XLSX 29 kb) Additional file 11: Table S10. List of 182 genes with higher expression levels in comparison 2Hb8 R/M69_F48 and were induced later in M69 at 96 hai. (XLSX 27 kb) Additional file 12: Table S11. List of 19 candidate genes for the 2Hb8 QTL. (XLSX 11 kb) Additional file 13: Table S12. List of 44 genes that were induced in the 6Hb7 R NIL at 48 hai and were induced in the susceptible parent Lacey at 96 hai. (XLSX 12 kb) Additional file 14: Table S13. List of 34 up-regulated genes in both R NILs when compared to respective susceptible recurrent parents at 48 hai. (XLSX 11 kb) Additional file 15: Table S14. List of lncRNAs identified from barley spike samples treated with water or *F. graminearum*. (XLSX 466 kb) Additional file 16: Table S15. Class codes of lncRNAs with regard to barley reference transcripts. (DOCX 12 kb) Additional file 17: Table S16. Number of differentially expressed lincRNAs (DELs) identified by all pairwise comparisons. (DOCX 11 kb) Additional file 18: Table S17. Lists of differentially expressed lncRNAs identified in all pairwise comparisons. (XLSX 131 kb) Additional file 19: Table S18. List of lncRNAs that showed co-induction with neighboring transcripts. (XLSX 8 kb)
