# Supplementary material for: Differential transcriptomic responses to Fusarium graminearum infection in two barley quantitative trait loci associated with Fusarium head blight resistance
Source: BMC Genomics. 2016 May 21;17:387. doi: 10.1186/s12864-016-2716-0 (PMC4875680; doi:10.1186/s12864-016-2716-0)
Supplement: Additional file 1: Figure S1. — Graphical genotypes of the 2Hb8 R and 6Hb7 R NILs. Figure S2. The R NILs accumulated less DON and ergosterol than the respective susceptible genotypes M69 and Lacey. Figure S3. Flowchart of the experimental setup and RNA-Seq analysis. Figure S4. Number of RNA-Seq reads generated for each sample that were filtered and uniquely mapped. Figure S5. Correlation coefficients of biological replicates used in RNA-Seq experiments. Figure S6. Correlation of RNA-Seq and qRT-PCR results of 13 genes and one lncRNA. Figure S7. MapMan visualization of FHB-responsive DEGs involved in general metabolic pathways in M69 at 48 hai and 96 hai. Figure S8. Categorization of induced (red) or repressed (blue) genes in Lacey at 48 and 96 h after F. graminearum inoculation. Figure S9. Categorization of induced (red) or repressed (blue) genes in the 6Hb7 R NIL compared to Lacey at 48 and 96 h after F. graminearum inoculation. Figure S10. Identification and characterization of lncRNAs from barley spike samples. Figure S11. Amino acid sequence alignment of three cysteine-rich receptor-like kinases using Jalview. (DOCX 6130 kb) [file 12864_2016_2716_MOESM1_ESM.docx]

BMC Genomics

**Differential transcriptomic responses to *Fusarium graminearum* infection in two barley quantitative trait loci associated with *Fusarium* head blight resistance**

Yadong Huang^1,#^, Lin Li^1, #^, Kevin P Smith^1^ and Gary J Muehlbauer^1,2,*^

^1^ Department of Agronomy and Plant Genetics, University of Minnesota, St. Paul, MN 55108

^2^ Department of Plant Biology, University of Minnesota, St. Paul, MN 55108

^#^ These authors contributed equally to this work

^*^ Correspondence: phone: 612-624-2755; Email: [muehl003@umn.edu](mailto:muehl003@umn.edu)

Supplementary Figures


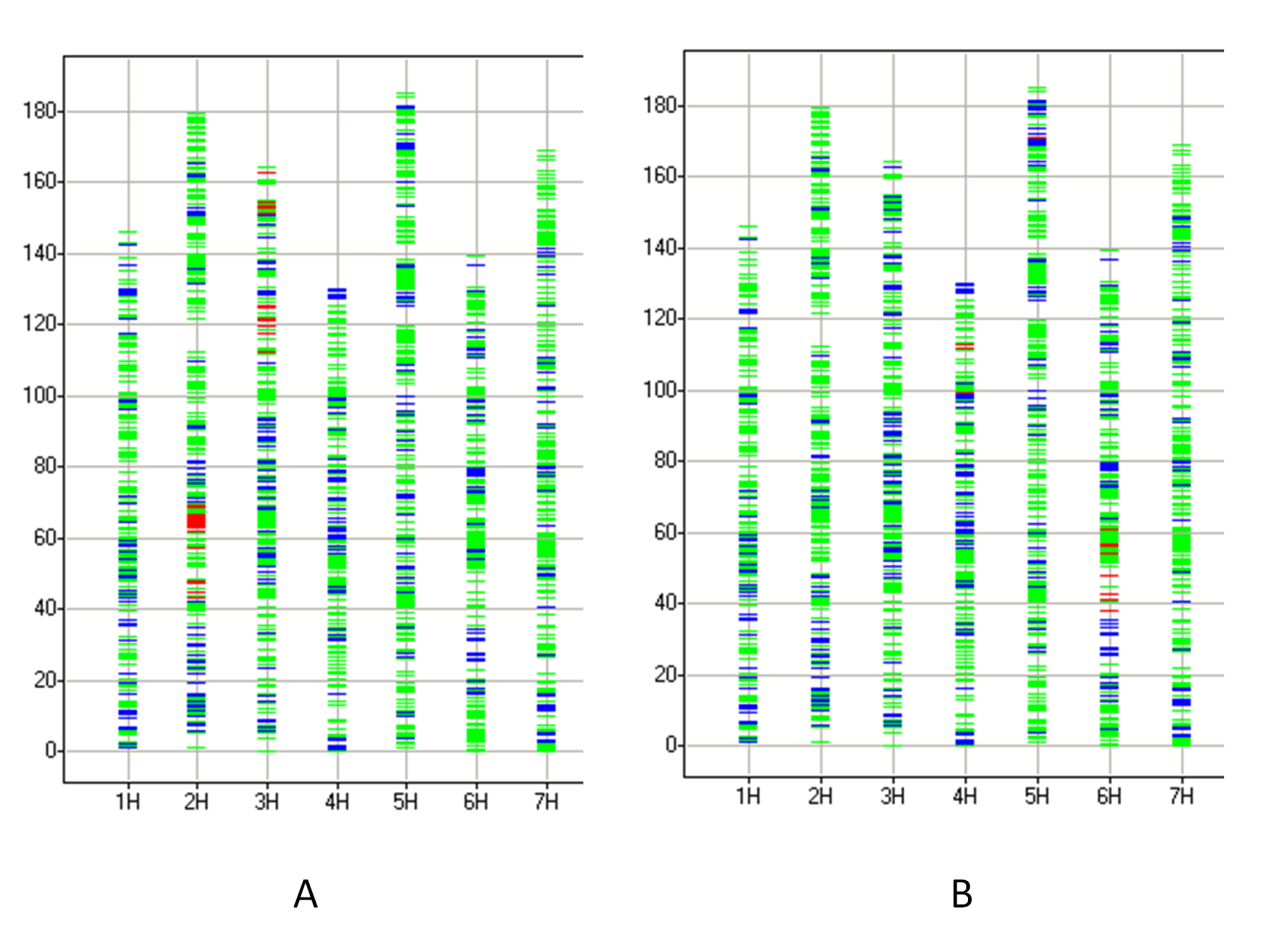


Figure S1. Graphical genotypes of the 2Hb8 R and 6Hb7 R NILs. (A) Genotype of the 2Hb8 R NIL. Chevron alleles are shown in red and M69 alleles in blue. The shared alleles between Chevron and M69 are in green. (B) Genotype of the 6Hb7 R NIL. Chevron alleles are in red and Lacey alleles in blue. The shared alleles between Chevron and Lacey are in green.


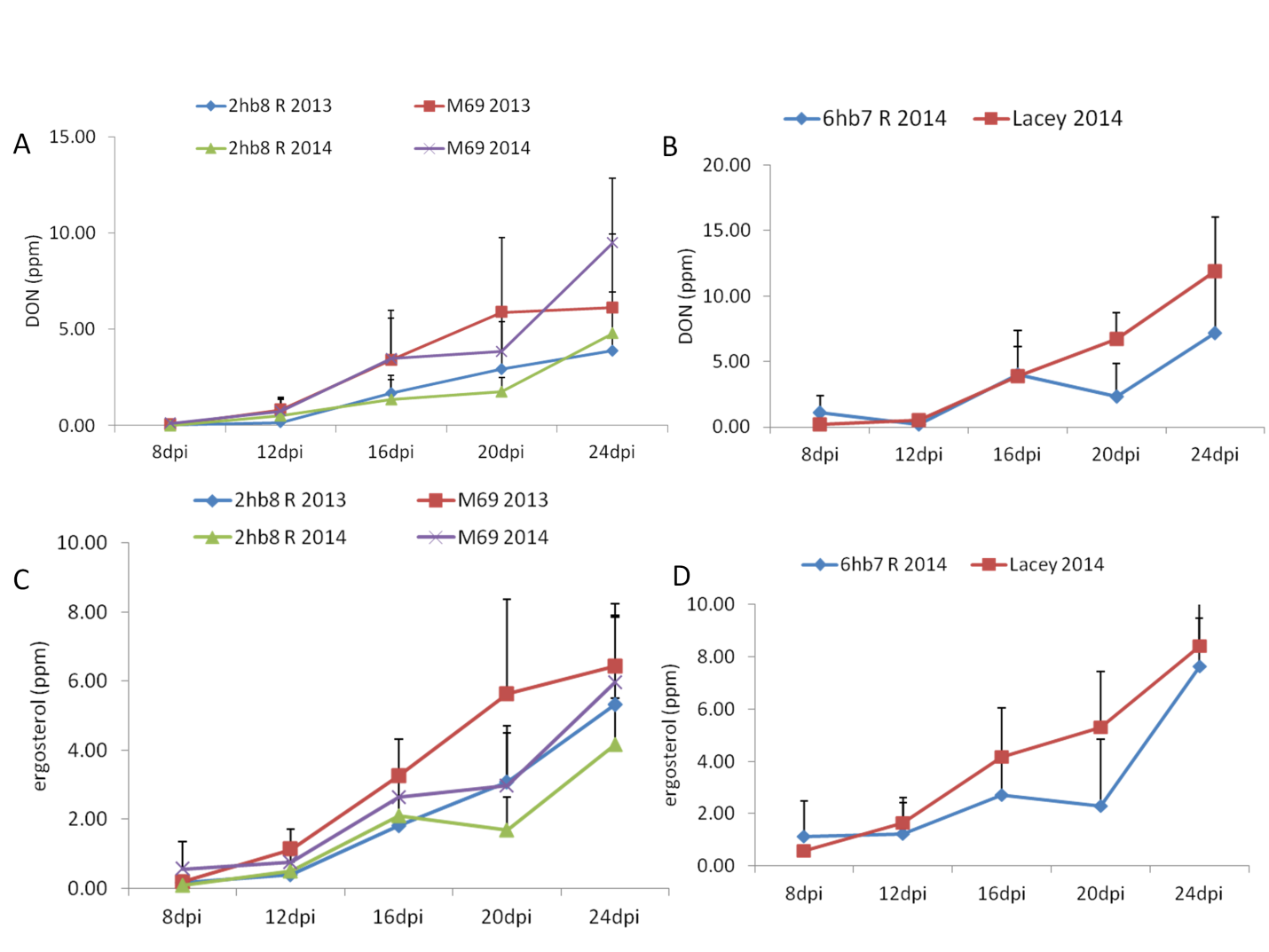


Figure S2. The R NILs accumulated less DON and ergosterol than the respective susceptible genotypes M69 and Lacey. Error bars indicate standard deviation. (A) DON accumulation in the 2Hb8 R NIL and M69 samples in 2013 and 2014. The difference between the R NIL and M69 was significant at 8 dpi (2014, *p*<0.05), 12 dpi (2013, *p*<0.05) and 24 dpi (2014, *p*<0.05). (B) DON accumulation in the 6Hb7 R NIL and Lacey samples in 2014. The difference was significant at 20 dpi (*p*<0.05). (C) Ergosterol accumulation in the 2Hb8 R NIL and M69 samples in 2013 and 2014. The difference was significant at 12 dpi (2013, *p*<0.05). (D) Ergosterol accumulation in the 6Hb7 R NIL and Lacey samples in 2014. There was no significant difference in this comparison.


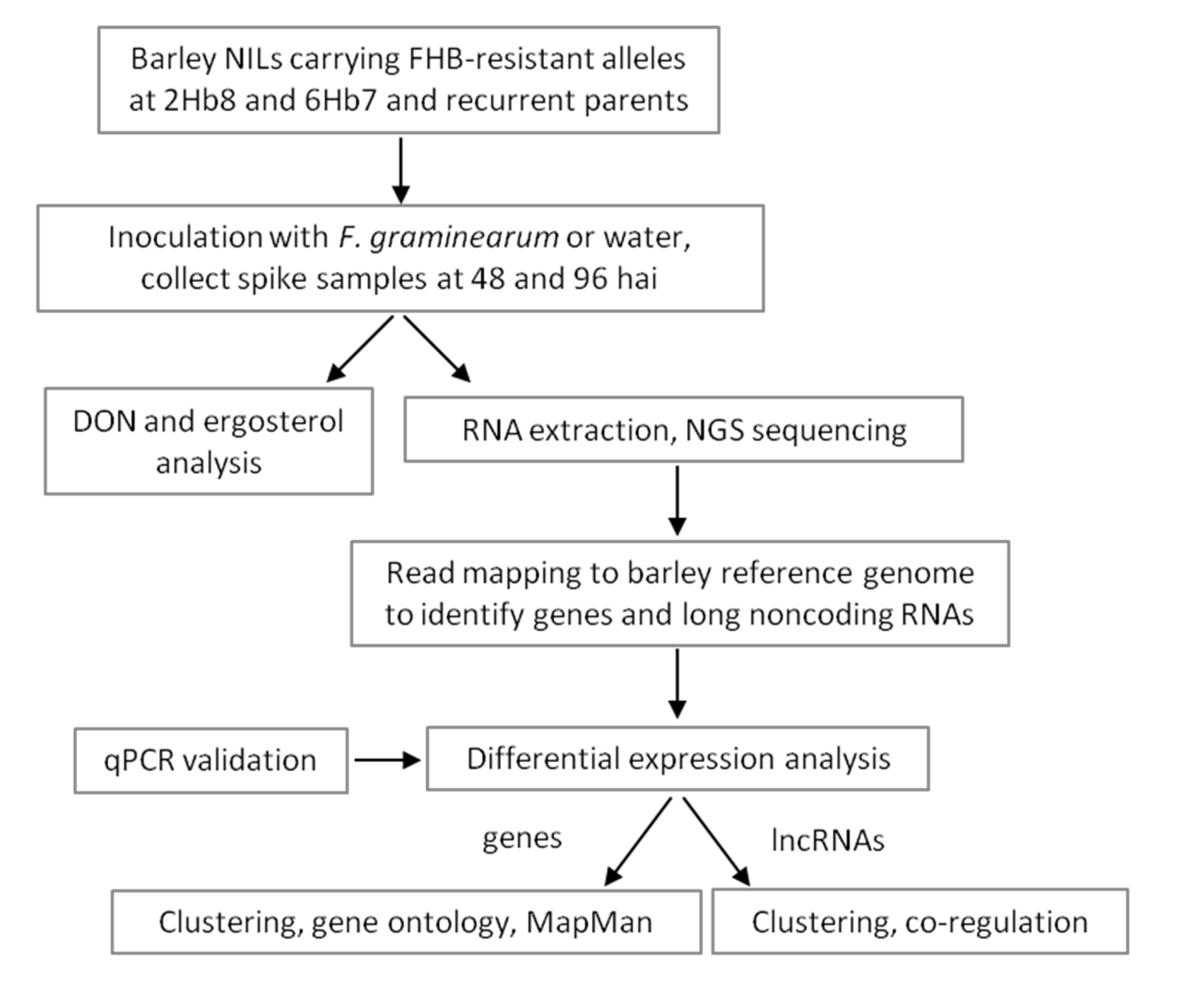


Figure S3. Flowchart of the experimental setup and RNA-Seq analysis.


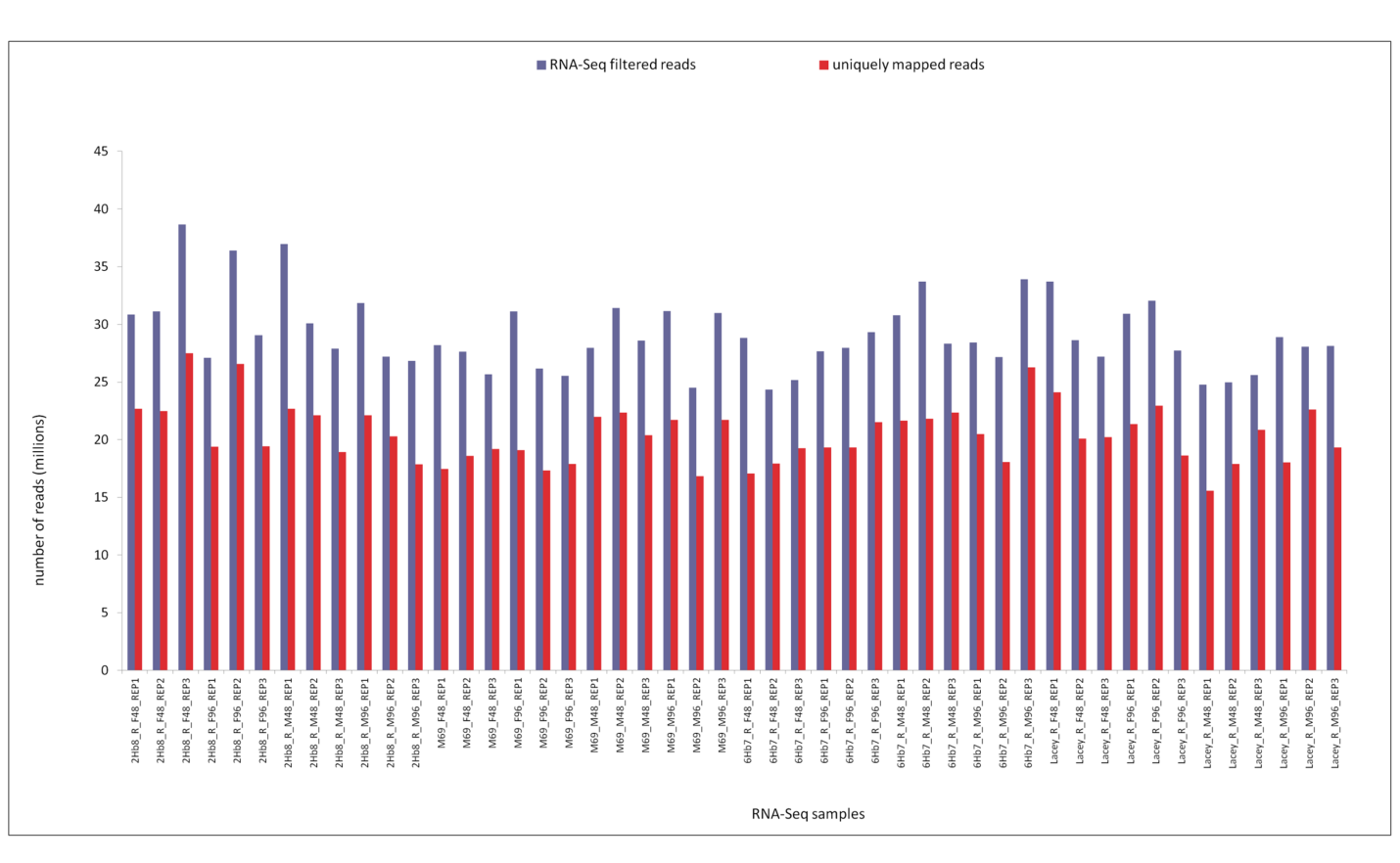


Figure S4. Number of RNA-Seq reads generated for each sample that were filtered and uniquely mapped.


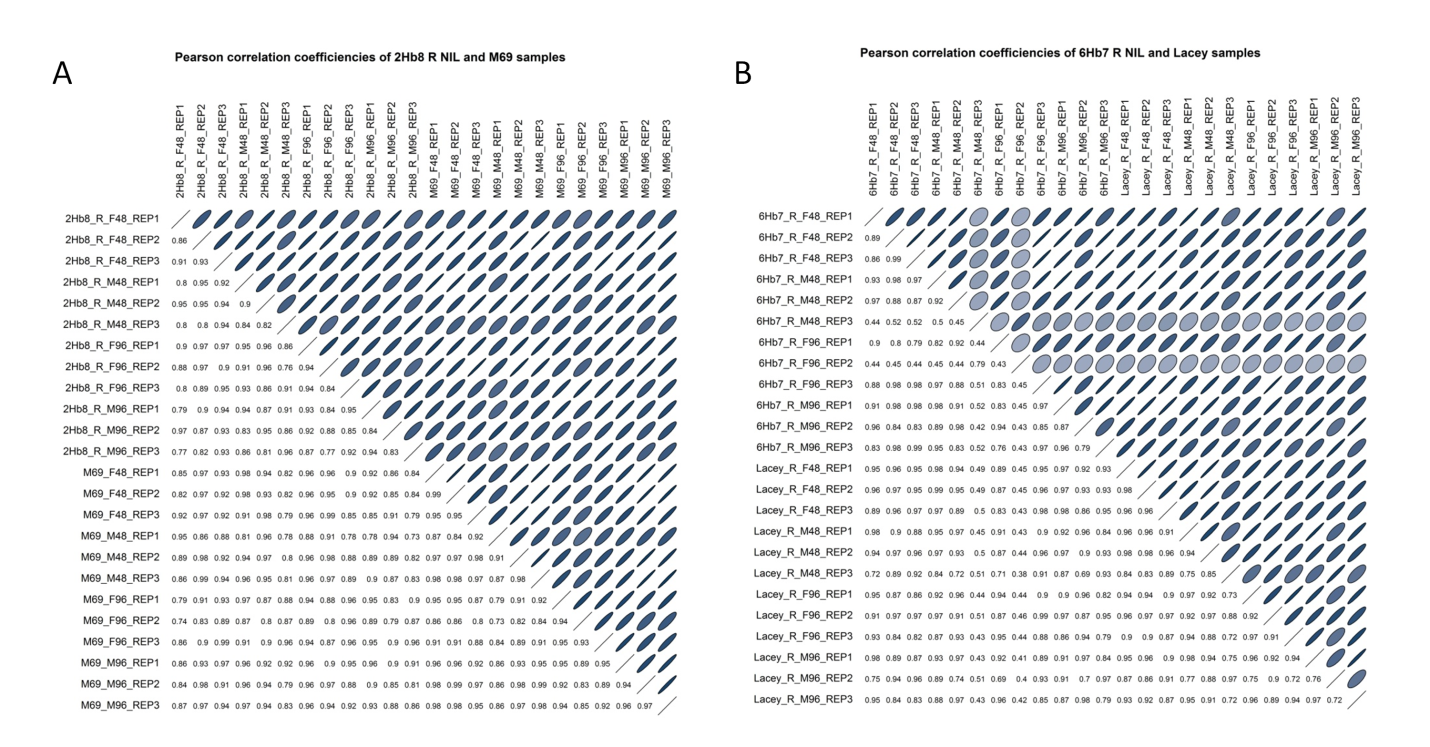


Figure S5. Correlation coefficients of biological replicates used in RNA-Seq experiments. (A) The 2Hb8 R NIL and M69 samples. (B) The 6Hb7 R NIL and Lacey samples. Replicates (6Hb7_R_M48_REP3 and 6Hb7_R_F96_REP2) with low correlation coefficients were removed from further analyses.


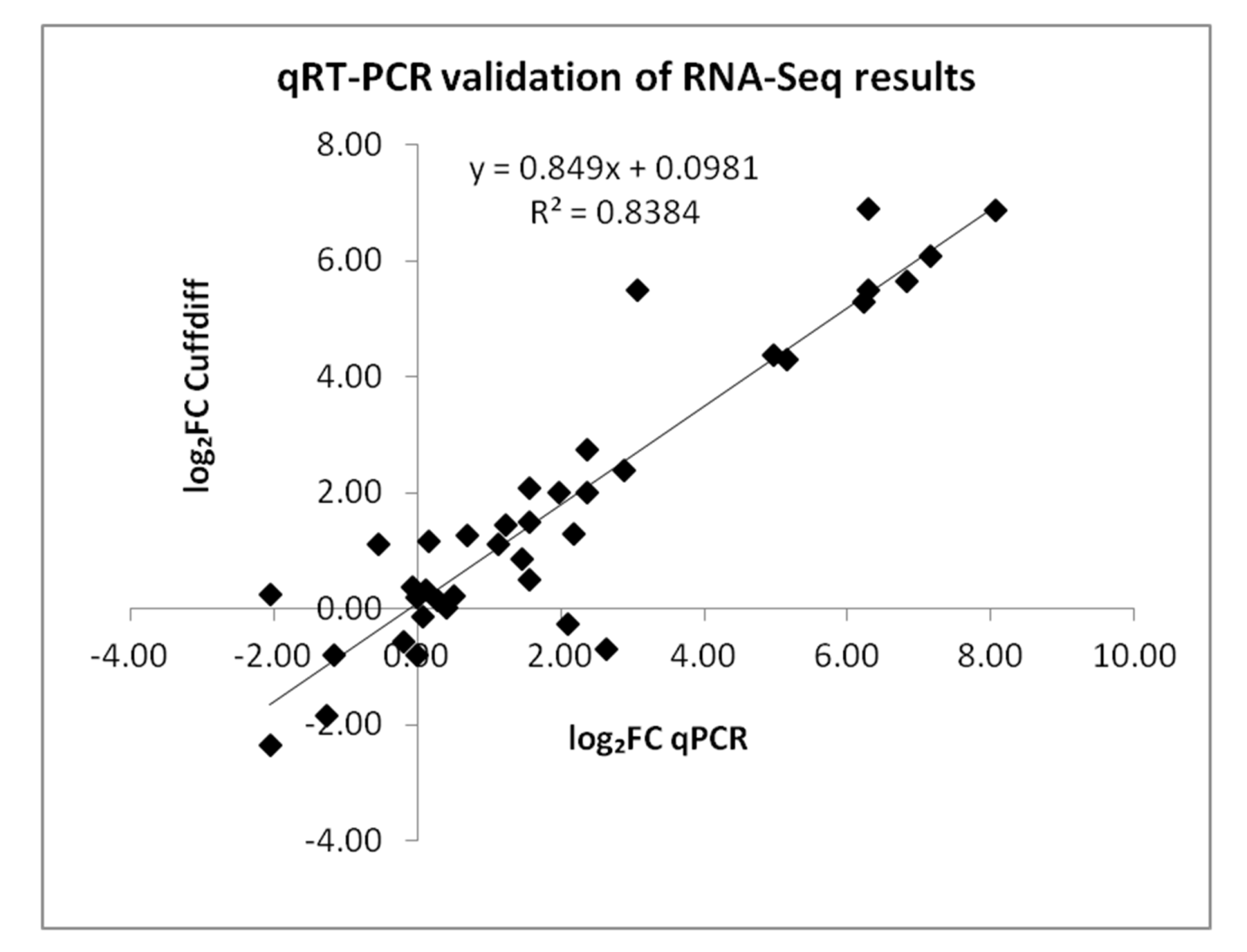


Figure S6. Correlation of RNA-Seq and qRT-PCR results of 13 genes and one lncRNA.


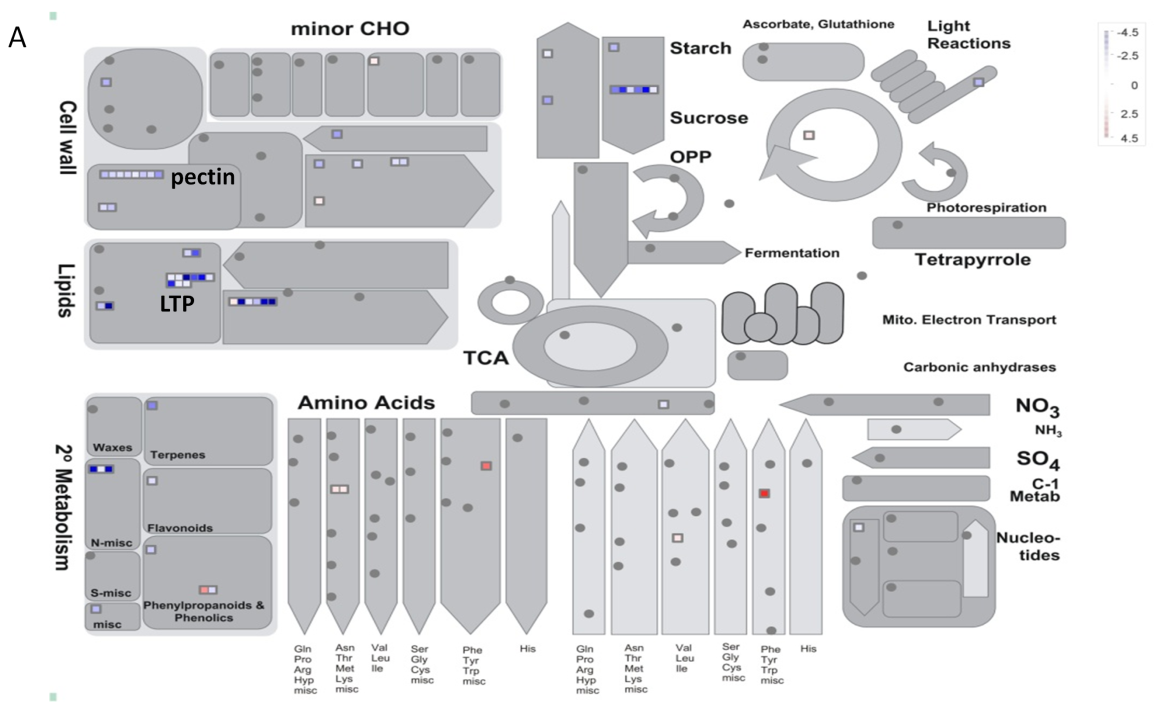


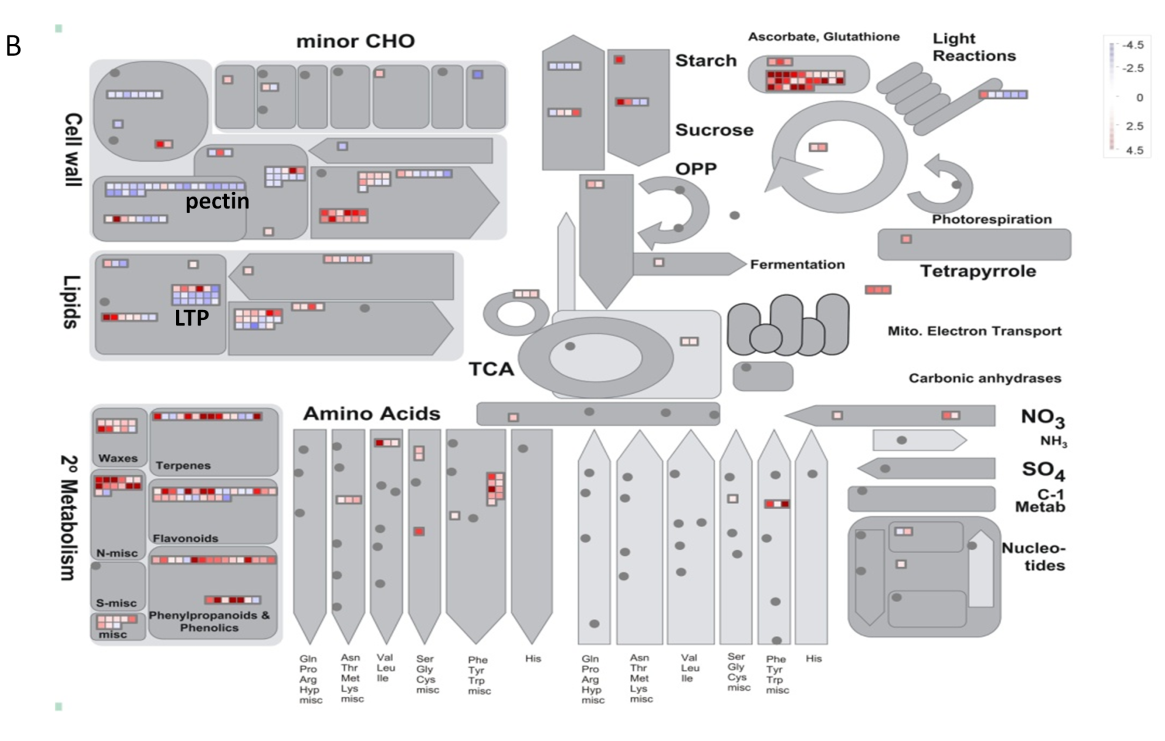


Figure S7. MapMan visualization of FHB-responsive DEGs involved in general metabolic pathways in M69 at 48 hai (A) and 96 hai (B). Note that the pectin enzymes and lipid transfer proteins (LTPs) were over-represented in genes down-regulated at both time points.


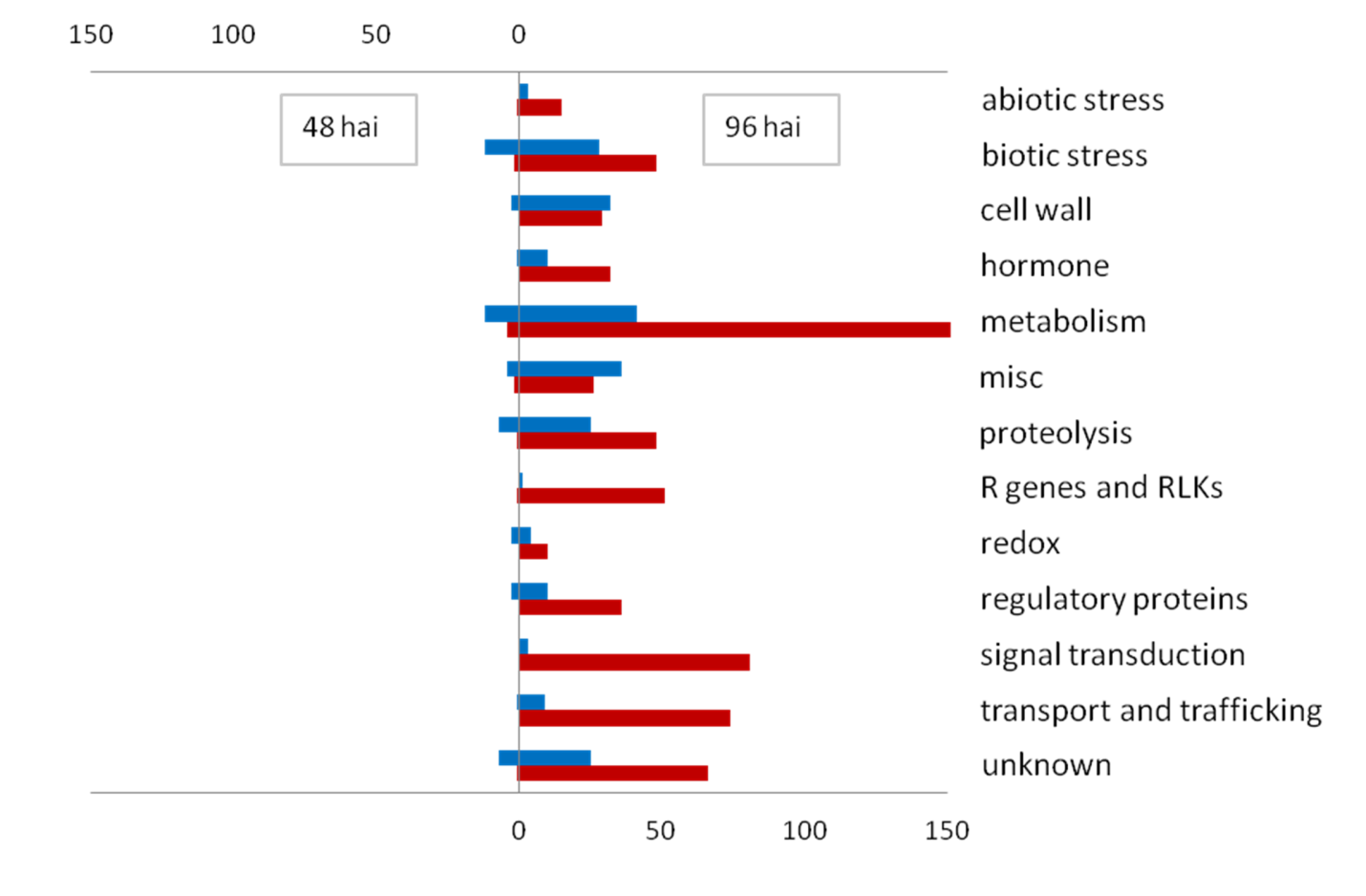


Figure S8. Categorization of induced (red) or repressed (blue) genes in Lacey at 48 and 96 h after *F. graminearum* inoculation.


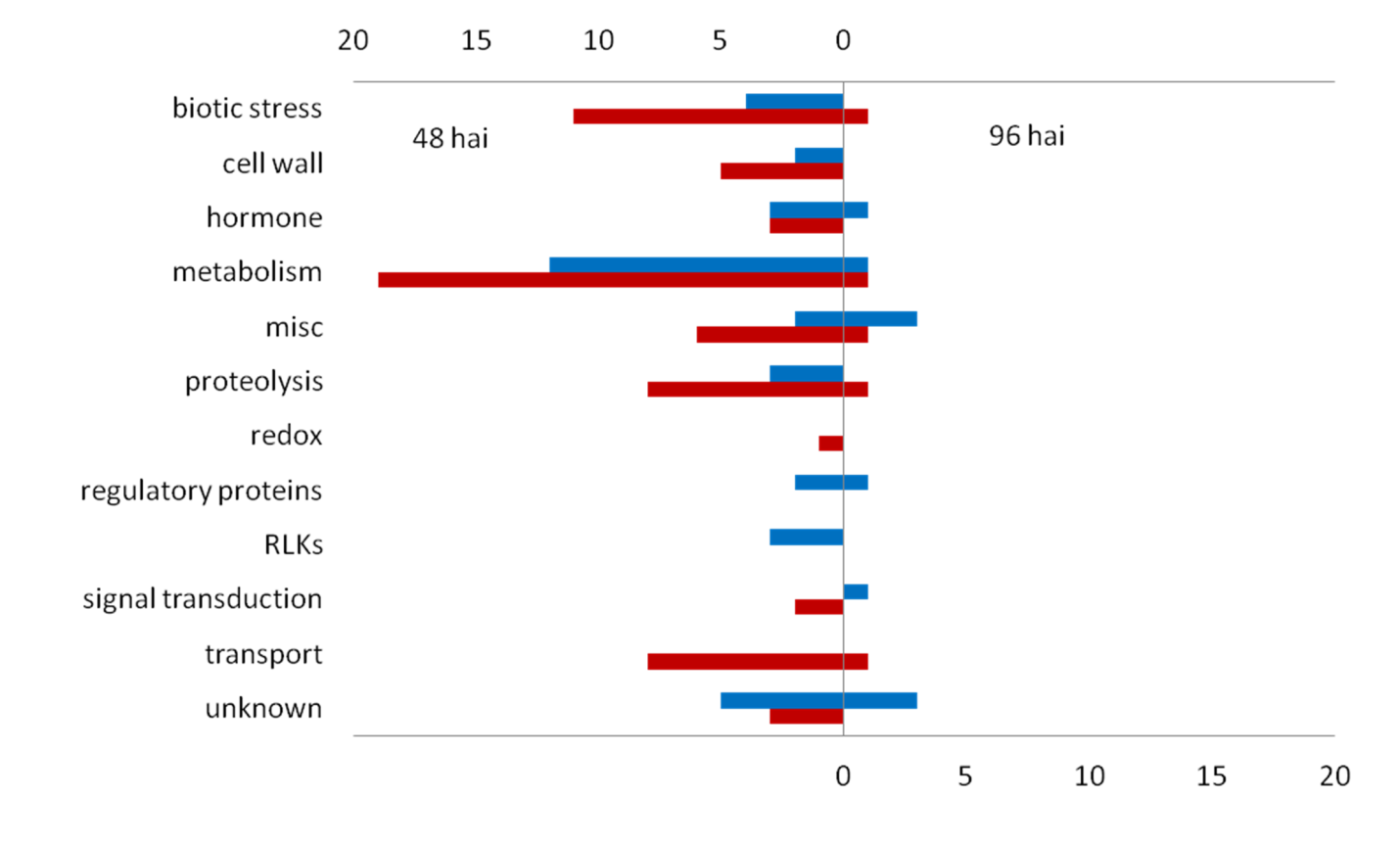


Figure S9. Categorization of induced (red) or repressed (blue) genes in the 6Hb7 R NIL compared to Lacey at 48 and 96 h after *F. graminearum* inoculation.


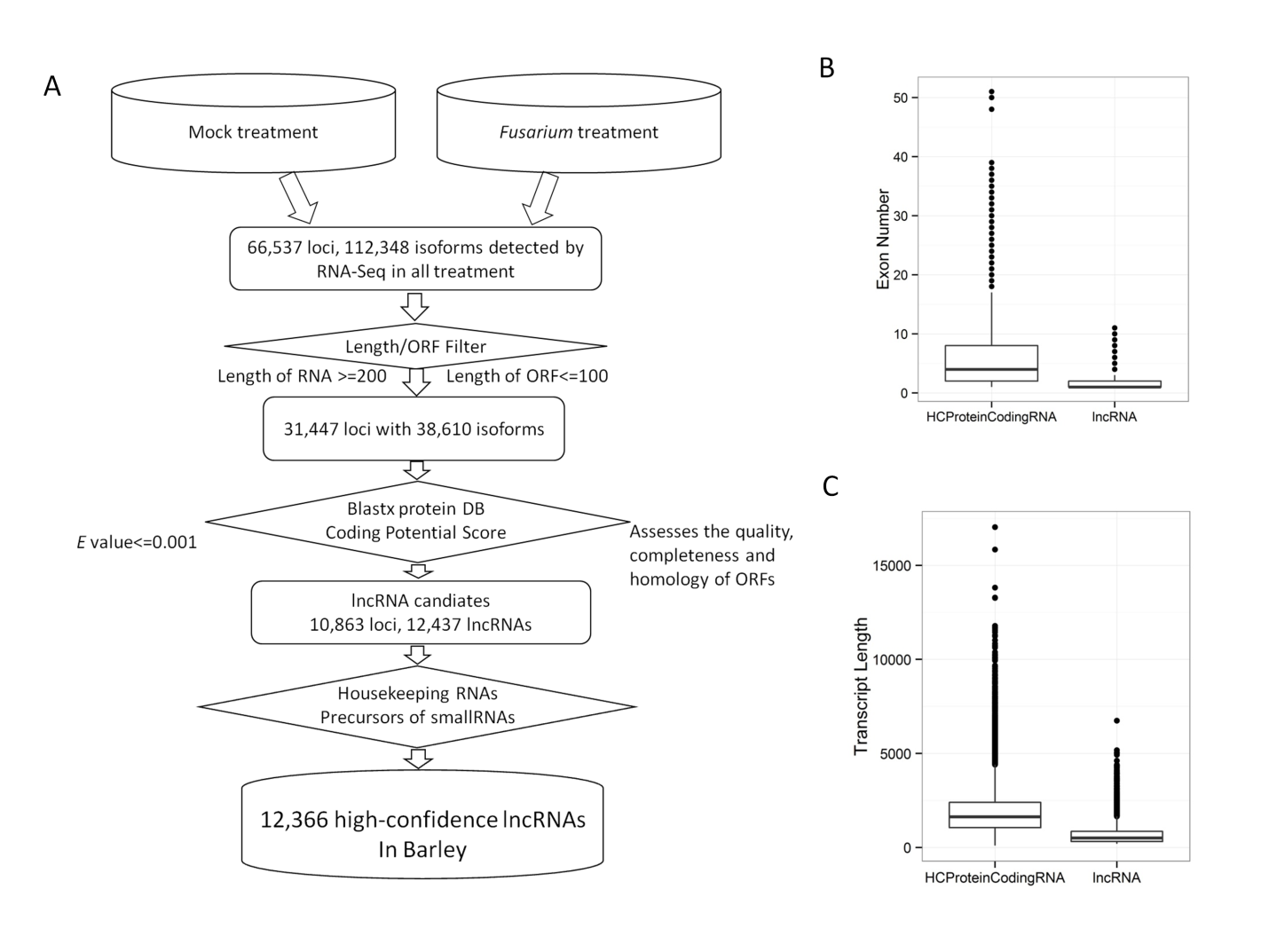


Figure S10. Identification and characterization of lncRNAs from barley spike samples. (A) Bioinformatics pipeline of barley lncRNA identification. (B) LncRNAs have fewer exons (mean=1.5) than protein-coding RNAs (mean=5.5), *p* <2.2e-16. (C) LncRNAs encode shorter transcripts (mean=0.7) than protein-coding RNAs (mean=1.9), *p* <2.2e-16.


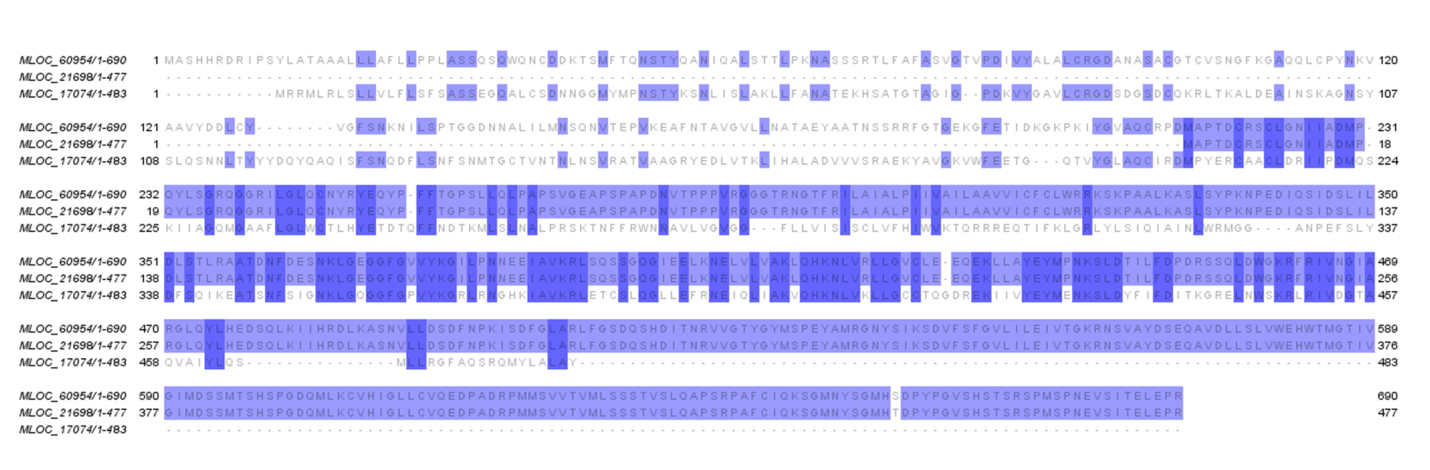


Figure S11. Amino acid sequence alignment of three cysteine-rich receptor-like kinases using Jalview.
