# Supplementary material for: Differential transcriptomic responses to Fusarium graminearum infection in two barley quantitative trait loci associated with Fusarium head blight resistance
Source: BMC Genomics. 2016 May 21;17:387. doi: 10.1186/s12864-016-2716-0 (PMC4875680; doi:10.1186/s12864-016-2716-0)
Supplement: Additional file 3: Table S2. — DON and ergosterol concentrations in the samples used for RNA-Seq. (DOCX 11 kb) [file 12864_2016_2716_MOESM3_ESM.docx]

| Sample | DON concentration (ppm) | | | | Ergosterol concentration (ppm) | | | |
| --- | --- | --- | --- | --- | --- | --- | --- | --- |
|  | F48 | F96 | W48 | W96 | F48 | F96 | W48 | W96 |
| 2Hb8 R NIL | 0 | 0.86±0.81 | 0 | 0 | 0 | 2.13±1.21 | 0 | 0 |
| M69 | 0 | 2.07±1.90 | 0 | 0 | 0 | 4.99±3.93 | 0 | 0.08±0.14 |
| 6Hb7 R NIL | 0 | 2.06±0.94 | 0 | 0 | 0 | 4.02±2.16 | 0 | 0 |
| Lacey | 0 | 2.08±0.36 | 0 | 0 | 0 | 4.11±1.68 | 0 | 0 |

Table S2 DON and ergosterol concentrations in the samples used for RNA-seq
