# Supplementary material for: Differential transcriptomic responses to Fusarium graminearum infection in two barley quantitative trait loci associated with Fusarium head blight resistance
Source: BMC Genomics. 2016 May 21;17:387. doi: 10.1186/s12864-016-2716-0 (PMC4875680; doi:10.1186/s12864-016-2716-0)
Supplement: Additional file 7: Table S6. — GO terms enriched in DEGs in susceptible parent M69 after F. graminearum inoculation. (DOCX 17 kb) [file 12864_2016_2716_MOESM7_ESM.docx]

Table S6 GO terms enriched in DEGs in susceptible parent M69 after *F. graminearum* inoculation
