# Supplementary material for: Differential transcriptomic responses to Fusarium graminearum infection in two barley quantitative trait loci associated with Fusarium head blight resistance
Source: BMC Genomics. 2016 May 21;17:387. doi: 10.1186/s12864-016-2716-0 (PMC4875680; doi:10.1186/s12864-016-2716-0)
Supplement: Additional file 16: Table S15. — Class codes of lncRNAs with regard to barley reference transcripts. (DOCX 12 kb) [file 12864_2016_2716_MOESM16_ESM.docx]

Table S15 Class codes of lncRNAs with regard to barley reference transcripts

| Class code | Number of transcript | Percentage (%) | Description |
| --- | --- | --- | --- |
| = | 234 | 1.89 | Complete match of intron chain |
| j | 30 | 0.24 | Potentially novel isoform (fragment): at least one splice junction is shared with a reference transcript |
| e | 7 | 0.06 | Single exon transfrag overlapping a reference exon and at least 10 bp of a reference intron, indicating a possible pre-mRNA fragment. |
| i | 40 | 0.32 | A transfrag falling entirely within a reference intron |
| o | 28 | 0.23 | Generic exonic overlap with a reference transcript |
| p | 253 | 2.05 | Possible polymerase run-on fragment (within 2Kbases of a reference transcript) |
| u | 11740 | 94.94 | Unknown, intergenic transcript |
| x | 34 | 0.27 | Exonic overlap with reference on the opposite strand |
| total | 12366 | 100 |  |
