# Supplementary material for: Differential transcriptomic responses to Fusarium graminearum infection in two barley quantitative trait loci associated with Fusarium head blight resistance
Source: BMC Genomics. 2016 May 21;17:387. doi: 10.1186/s12864-016-2716-0 (PMC4875680; doi:10.1186/s12864-016-2716-0)
Supplement: Additional file 17: Table S16. — Number of differentially expressed lincRNAs (DELs) identified by all pairwise comparisons. (DOCX 11 kb) [file 12864_2016_2716_MOESM17_ESM.docx]

Table S16 Number of differentially expressed lincRNAs (DELs) identified by all pairwise comparisons

|  | RNA-Seq datasets comparison | number of DELs | | | |
| --- | --- | --- | --- | --- | --- |
|  |  | 48 hai | | 96 hai | |
|  |  | up | down | up | down |
| 2Hb8 | R NIL Fusarium inoculated – R NIL mock inoculated | 12 | 40 | 135 | 45 |
|  | M69 Fusarium inoculated – M69 mock inoculated | 10 | 37 | 216 | 59 |
|  | R NIL Fusarium inoculated – M69 Fusarium inoculated | 135 | 96 | 79 | 105 |
|  | R NIL mock inoculated – M69 mock inoculated | 120 | 64 | 162 | 181 |
| 6Hb7 | R NIL Fusarium inoculated – R NIL mock inoculated | 53 | 9 | 57 | 26 |
|  | Lacey Fusarium inoculated – Lacey mock inoculated | 13 | 29 | 122 | 26 |
|  | R NIL Fusarium inoculated – Lacey Fusarium inoculated | 66 | 37 | 35 | 54 |
|  | R NIL mock inoculated – Lacey mock inoculated | 41 | 60 | 55 | 18 |
